# Mathematics Partial Associations with Subject-Specific Science Confidence: Evidence from 11 Education Systems in TIMSS 2023

**DOI:** 10.3390/bs16101924

**Published:** 2026-10-17

**Authors:** Georgios D. Sideridis, Mohammed Alghamdi

**Affiliations:** 1 Boston Children’s Hospital, Harvard Medical School, Boston, MA 02115, USA; 2 Department of Self-Development Skills, King Saud University, Riyadh 11451, Saudi Arabia; mhalghamdi@ksu.edu.sa

**Keywords:** dimensional comparison theory, internal/external frame of reference model, academic self-concept, domain specificity, plausible values, TIMSS

## Abstract

Dimensional comparison theory holds that achievement in one school subject is positively associated with self-concept in that subject and negatively associated with self-concept in dissimilar subjects. Applications within the science domain have characterized biology, chemistry, physics, and earth science relative to one another while omitting mathematics from the estimated model. The present study estimated the partial association of mathematics achievement with each of four subject-specific science confidence scales, conditional on achievement in all four science content domains, across the 11 TIMSS 2023 Grade 8 education systems administering subject-specific questionnaires. Because the achievement indicators are strongly intercorrelated, each coefficient is interpreted as an association with the residual variation in mathematics achievement that survives the conditioning, which retains 30% to 52% of its variance by system, and not as a measure of mathematics-specific competence. Zero-order associations were positive for all four subjects; conditioning on science achievement reversed the sign for biology under every parameterization examined. The conditional association was negative for biology confidence (β = −0.158, 95% CI [−0.224, −0.093], Holm-adjusted *p* = 0.001) and positive for physics confidence (β = 0.093, 95% CI [0.041, 0.146], Holm-adjusted *p* = 0.009). The chemistry association was positive but specification-dependent (β = 0.066, *p* = 0.035) and is reported as inconclusive, and the earth science association was not distinguishable from zero. Forming the change in each within-science cross-path inside every replicate and plausible value, both prespecified physics–chemistry paths were attenuated by the mathematics anchor (Δ = −0.027, unadjusted *p* = 0.012, Holm-adjusted *p* = 0.024; Δ = −0.014, unadjusted *p* = 0.040, Holm-adjusted *p* = 0.040), while other cross-paths were amplified or reversed; a covariance-matched placebo anchor established, algebraically and numerically, that the expected mechanical attenuation is zero. Forward and reverse associations did not differ, though the design excludes only asymmetries larger than about 0.08 to 0.13. Between-system heterogeneity was the most consistent finding (*I*^2^ = 56–87%): prediction intervals included zero for all four associations, and while biology was the most negative subject in 11 of 11 systems and the biology–physics contrast held in direction in every system, the complete four-subject ordering recurred in 7 of 11 and had a probability above 0.50 in only 4. Subject-specific science confidence relates heterogeneously to mathematics achievement, and aggregation across science subjects can obscure associations of opposing sign.

## 1. Introduction

The domain specificity of academic self-concept constitutes one of the more replicated regularities in educational measurement (Marsh & Shavelson, 1985; Möller et al., 2020). Students do not maintain a unitary appraisal of scholastic competence; rather, they maintain separable and only moderately intercorrelated appraisals across curricular domains (Arens et al., 2021; Marsh & Shavelson, 1985; Möller et al., 2020). The substantive consequence of this multidimensionality is expressed in the internal/external frame of reference (I/E) model, in which achievement in a domain is positively associated with self-concept in the matching domain and negatively associated with self-concept in non-matching domains (Marsh, 1986), and it is interpreted through dimensional comparison theory, in which comparisons across domains yield contrast when domains are appraised as dissimilar and assimilation when they are appraised as similar (Möller, 2024; Möller & Marsh, 2013). Both are set out in Section 2, which also reviews the extension of the model within the science domain and the measurement constraints that bear on it.

Where biology, chemistry, and physics are delivered as discrete subjects, students maintain separable self-concepts for them (Jansen et al., 2014; Saß & Kampa, 2019; Schroeders & Jansen, 2022), and the dimensional structure of those self-concepts varies with the composition of the courses in which students are enrolled (Jansen et al., 2019). The substantive studies that have estimated I/E models within science are reviewed in Section 2.2. What matters for the present purpose is a feature they share: mathematics, one of the two termini of the academic continuum on which the interpretation of these models rests, is not among the estimated predictors.

Concurrent work has examined confidence in the present assessment cycle. Rutkowski et al. (2026) documented the descriptive relation between achievement and self-confidence in mathematics and in science across the TIMSS 2023 systems, establishing the magnitude of the association at the level of the two aggregate domains. The present study is complementary rather than overlapping in three respects: it disaggregates science into its four constituent subjects, it estimates partial rather than bivariate associations by conditioning each subject on achievement in all remaining domains, and it synthesizes system-specific estimates meta-analytically so that heterogeneity is quantified rather than averaged over.

### 1.1. The Interpretive Gap

The findings summarized above are not in dispute. What is at issue is the inferential status of the interpretation placed upon them. Guo et al. (2017) concluded that “students perceive earth science to be relatively similar to other science domains, implying that earth science would be located between physics/chemistry and biology in the verbal-math continuum” (p. 88). The same report states, however, that “mathematics and verbal skills are posited as the endpoints of the academic continuum were not considered in this study” (p. 88). Statements regarding the location of a science subject therefore rest on the configuration of within-science cross-paths interpreted through a theoretical prior, rather than on any estimated relation to a terminus.

Two consequences follow. First, the assimilation reported between physics and chemistry is confounded with content. Physics and chemistry share quantitative demands to a degree that biology does not, a gradient documented in curricular regulation and in subject-specific research and reviewed in Section 2.2 (Ofqual, 2020; Palmgren et al., 2025; Ye et al., 2024); the corresponding evidence for earth science could not be established, and no claim is advanced about its position. A positive association between physics achievement and chemistry self-concept is equally consistent with dimensional comparison between adjacent domains and with both subjects functioning partly as indicators of quantitative competence. Second, the literature sustains divergent characterizations of biology self-concept: as behaving in a mathematics-like manner (Arens et al., 2018), as aligned with neither terminus (Jansen et al., 2015; Marsh et al., 2015b), and as verbal-like (Guo et al., 2017). These characterizations, summarized in Table 1, derive from different national samples, instruments, tracks, and model specifications, and are not necessarily contradictory; adjudication nonetheless requires a common anchor estimated in multiple systems.

The term *anchor* is used throughout in a specific and deliberately narrow sense. It denotes an additional conditioning predictor entered alongside the science achievement scores in a single regression equation: mathematics achievement is held constant in the estimation of the science cross-paths, and its own partial coefficient is read as the quantity of interest. It does not denote a moderator, and no interaction between mathematics achievement and any other predictor is estimated or implied. Nor does it denote an anchor in the psychometric sense of a common item or common person linking two scales to a shared metric. It is equally essential to state what such an anchor can and cannot deliver. A standardized partial regression coefficient relating mathematics achievement to a science confidence scale, conditional on four highly correlated science achievement indicators, is an association with the component of mathematics achievement that those indicators do not explain. It is not a latent coordinate, it does not identify a position between a mathematical and a verbal terminus, and it should not be described as capturing what is specific to mathematics: the residual is defined by the conditioning set, not by the construct. TIMSS administers no verbal achievement assessment at Grade 8, so no verbal anchor is available. The present study accordingly estimates mathematics-anchored partial associations and interprets them as such throughout.

### 1.2. Statement of Importance

The question carries consequences beyond the theoretical literature. Intervention designers, curriculum authorities, and assessment agencies routinely treat science self-concept as a unitary target, and international assessments report aggregate science motivation for the majority of participating systems. If subject-specific science confidence scales relate to mathematics achievement with associations of opposing sign, then an aggregate of those scales averages heterogeneous relations, and inferences drawn from the aggregate will not transfer uniformly to its constituents. Establishing whether such heterogeneity exists, and how far it generalizes across education systems, is a precondition for the interpretation of aggregate science motivation measures rather than a refinement of them.

A second consideration concerns method. Dimensional comparison effects within the mathematics and science domains are small: Möller et al. (2020), synthesizing 505 datasets, reported associations near zero when both subjects belonged to that domain, and stronger associations for school grades than for standardized achievement. Any study using standardized achievement to characterize within-science comparison therefore operates under conditions unfavorable to detection, and the credibility of its conclusions depends on the uncertainty attached to each estimate rather than on the point estimates alone.

### 1.3. Goals of the Present Study

The main goal of the present study was to estimate the partial association of mathematics achievement with confidence in biology, earth science, chemistry, and physics, conditional on achievement in all four science content domains, across the education systems administering subject-specific questionnaires in TIMSS 2023. A secondary goal was to determine whether the within-science cross-paths reported by Guo et al. (2017) are attenuated by the introduction of the mathematics anchor beyond the attenuation that the addition of any correlated predictor produces mechanically. A third goal was to characterize the extent of between-system heterogeneity in these associations and to establish which conclusions survive it.

The article is organized as follows. Section 2 reviews the I/E model and the similarity gradient, the evidentiary status of within-science dimensional comparison, the curricular and measurement constraints that bear on the design, and derives the four hypotheses from that review. Section 3 sets out the estimand, its identification limits, and the estimation and synthesis procedures. Section 4 reports the applied analysis of the TIMSS 2023 Grade 8 data, beginning with the nested conditioning sequence that fixes the interpretation of the principal coefficients. Section 5 considers the implications, the alternative explanations the design cannot exclude, and the limitations.

## 2. Literature Review

### 2.1. The Internal/External Frame of Reference Model

Marsh (1986) proposed that academic self-concept in a subject is formed through two comparisons. In the external comparison, students compare their achievement with that of classmates, producing a positive relation between achievement and self-concept within the subject. In the internal comparison, students compare their achievement in that subject with their own achievement in others, producing a negative relation between achievement in one subject and self-concept in another. Meta-analytic evidence supports the pattern (Möller et al., 2020), and its cross-cultural generalizability has been examined in TIMSS across twelve countries at two grades (Marsh et al., 2015a), across 28 countries (Chiu, 2008), in combination with the big-fish-little-pond effect (Chiu, 2012), and in the combined PIRLS and TIMSS Grade 4 data (Guo et al., 2018). Each of these cross-national applications treated science as a single aggregate domain.

### 2.2. Dimensional Comparison Within the Science Domain

Two lines of evidence establish internal differentiation of the science domain. The first is structural. Guo et al. (2017) rejected a second-order model in which a global science self-concept generated four subject-specific factors (CFI = 0.852, TLI = 0.820, against CFI = 0.963 for the correlated-factors representation), with latent correlations among the four subject self-concepts of 0.28 to 0.42. The second is the extension of the I/E model itself, described above. Two features of this evidence base bear on what can be claimed: the associations are small, and they are estimated in a small number of systems.

The premise that school science subjects differ in quantitative demand, which the interpretation in Section 1.1 and Hypothesis 3 both rely on, is supported by curricular and regulatory evidence rather than assumed. In England the statutory subject-level requirements set a graded minimum weighting of marks for Level 2 mathematical skills across the three sciences: at least 10% in biology, 20% in chemistry, and 40% in physics (Ofqual, 2020). Discipline-specific evidence points the same way, with systematic review establishing the constitutive role of mathematics in physics education (Palmgren et al., 2025) and detailed analysis documenting the mathematical modelling load in chemistry problem solving (Ye et al., 2024). Two qualifications are necessary. The gradient is documented for biology, chemistry, and physics; comparable evidence for earth science was not located, so the claim is advanced for the physical sciences against biology and is left open for earth science. And the evidence is national: curricular quantitative demand is a property of a system’s syllabus rather than of a subject in the abstract, which is one reason the associations estimated here are expected to vary across systems rather than to replicate at a common magnitude.

### 2.3. Achievement Measurement as a Substantive Constraint

The science content-domain achievement scales are strongly intercorrelated. Guo et al. (2017) reported latent correlations of 0.77 to 0.81 and observed that their data furnished “relatively weak support for the domain specificity of achievement scores” (Guo et al., 2017, Appendix D). In TIMSS 2023 the intercorrelations are higher still: across the systems analyzed here, median correlations among the four science content-domain plausible values ranged from 0.84 to 0.89, and mathematics correlated 0.76 to 0.78 with each science domain.

This matters because the quantity of interest is a partial coefficient. Under pronounced collinearity, partial coefficients are sensitive to differential reliability among predictors, and reliability differs across TIMSS content domains by construction: the Grade 8 assessment contributed 113 items to biology, 77 to physics, 66 to earth science, and 62 to chemistry, against 318 for science overall, and the technical documentation cautions that the subscales are “based on only a subset of the assessment items, making them somewhat less robust” than the total (Fishbein et al., 2025). A further distinction requires emphasis: the achievement indicators are content-domain scores derived from a single matrix-sampled science assessment, whereas the outcomes concern confidence in school subjects. Content domains and enrolled courses are related but not identical, and the achievement indicators should not be treated as direct measures of course-specific performance.

Plausible values do not resolve these issues. They represent uncertainty in population proficiency under the TIMSS scaling and conditioning model rather than a correction for measurement error in an individual score, and their use secures appropriate uncertainty for population-level inference rather than unbiased individual-level predictors (Fishbein et al., 2025).

A second constraint concerns the comparability of the outcome measures rather than the predictors. The confidence scales are mixed-worded, and inconsistent responding to mixed-worded scales in international assessments is both common and systematically related to achievement, age, and sex (Steinmann et al., 2024), varies with assessment mode and grade (Konstantinidou & Michaelides, 2025), and has been documented across countries in the most recent PISA cycle (Michaelides et al., 2026). Whether noncognitive scales in TIMSS and PISA support cross-country comparison at all has been questioned directly (He et al., 2019), and formal invariance testing of self-concept scales in these surveys frequently fails at the scalar level (Ding et al., 2023; Wurster, 2022). A design that compares associations across education systems therefore cannot treat the released scale scores as interchangeable across systems without diagnosis, and Section 4.2 reports that diagnosis.

### 2.4. Hypotheses

The four hypotheses follow from the three strands reviewed above, and each is derived here before it is stated. Hypothesis 1 follows from the similarity gradient of Section 2.1 together with the curricular evidence of Section 2.3. If dimensional comparison contrasts subjects appraised as dissimilar and assimilates subjects appraised as similar, and if the quantitative demands of school science subjects are graded as the regulatory and curricular evidence indicates, then conditioning on science achievement should leave mathematics achievement negatively associated with confidence in the subjects of lower quantitative demand and null or positively associated with confidence in those of higher demand. Hypothesis 2 follows from Jansen et al. (2015), who reported that achievement in the sciences bore no relation to self-concepts in other subjects while the converse did not hold; extending that design to disaggregated science yields a directional prediction. Hypothesis 3 follows from the confound identified in Section 2.2: if the physics–chemistry assimilation reported by Guo et al. (2017) is partly carried by shared quantitative content rather than by comparison between adjacent domains, then introducing an explicit mathematics anchor should reduce it. Hypothesis 4 requires a more careful derivation than it was originally given, because dimensional comparison theory does not by itself predict cross-system invariance. The theory specifies a process operating within individuals, and the reviewed evidence documents substantial variation across national samples, instruments, tracks, and course structures. What the theory does support is a prediction about *sign*: if the ordering of subjects on the similarity gradient is a property of their content rather than of the system that teaches them, the *direction* of the contrast between the extreme subjects should recur wherever the subjects are taught separately, even where its magnitude does not. Invariance of the complete four-subject ordering is a considerably stronger claim, and invariance of magnitudes stronger still. Hypothesis 4 is therefore stated below as a graded set of claims rather than as a single proposition, and is evaluated claim by claim. Hypotheses 1 to 4 are confirmatory. The comparison of cross-paths not involving physics and chemistry, the analyses of individual within-science cross-paths, and the exploration of mathematics content domains are exploratory and are labelled as such in the Results.

**Hypothesis 1.**  *The partial association of mathematics achievement with subject confidence is negative for biology and earth science and null or positive for chemistry and physics, conditional on achievement in all four science content domains. This was evaluated both through the four individual associations and through two prespecified ordering contrasts*.

**Hypothesis 2.**  *The associations are asymmetric, such that mathematics achievement relates to science confidence more strongly than science achievement relates to mathematics confidence, extending Jansen et al. (2015) to disaggregated science*.

**Hypothesis 3.**  *The physics–chemistry cross-paths reported by Guo et al. (2017) are attenuated upon introduction of the mathematics anchor. The attenuation is assessed against a benchmark of zero, which Section 3.5 shows to be the expected change produced by adding a covariance-matched predictor that carries no conditional association with the outcome, and which a placebo anchor is used to verify numerically*.

**Hypothesis 4.**  *Cross-system regularity in the ordering of the four subjects is graded. (a) The sign of the biology–physics contrast is invariant across education systems. (b) Biology occupies the most negative position of the four in every system. (c) The complete rank order of the four subjects is reproduced in every system. (d) The magnitudes of the four associations are homogeneous across systems. Claims (a) and (b) are evaluated both by tabulation and by the probability of the corresponding ordering event under each system’s estimated sampling distribution; claim (c) likewise; claim (d) by the heterogeneity statistics of the meta-analysis. The prespecified contrasts δ_1_ and δ_2_ bear on (a) and on the grouped comparison, and do not by themselves encode the complete ordering, which is why (c) is evaluated separately*.

## 3. Method

### 3.1. Data and Sampling Design

Data were obtained from the TIMSS 2023 Grade 8 International Database (Fishbein et al., 2025). TIMSS employs a two-stage stratified cluster design in which schools are sampled with probability proportional to size within explicit strata and intact classrooms are sampled within schools. Achievement was assessed under a matrix-sampled booklet design and reported as five plausible values per scale. Systems delivering science as an integrated subject received a single set of science attitude items; systems delivering science as discrete subjects received parallel item sets for biology, earth science, chemistry, and physics. Only the latter supported the present analysis.

### 3.2. Participants, Exclusions, and the Target Population

The 50% coverage threshold requires justification, since no conventional standard governs it. It was set before estimation on two grounds. Substantively, the block filter records current enrolment, so coverage below one half means that the majority of sampled students are not enrolled in the subject and the resulting estimate would describe a minority subgroup whose composition differs markedly from the sampled population; the common sample in such a system would be smaller still, because it requires valid data on all four subjects simultaneously. Statistically, retention below 50% in one subject implies a common sample too small to support the within-system contrasts that span equations. The threshold is nonetheless a convention rather than a criterion, and its consequences are bounded and reported. Neither excluded system is a borderline case: Romania returned 38.0% coverage on the earth science block, and Malta returned 21.5% on chemistry and 33.5% on biology. Nor is any retained system close to the cut, the lowest block coverage among the eleven being 73.5% (physics in Finland). Any threshold between 44% and 73% would therefore produce exactly the same analytic set, and the decision is not one on which the composition of the sample turns.

The separate-science questionnaire routes respondents by subject: each block is preceded by a filter item, and a negative response suppresses the block. The resulting absence is coded as *logically not applicable* and is generated by current enrollment rather than by nonresponse. Two analytic samples were therefore defined and both are reported. The outcome-specific sample retains every eligible case for each outcome and is primary. The common sample requires valid data on all five confidence scales and both covariates; it is necessary for contrasts spanning equations, and it defines a population of students concurrently enrolled in all four science subjects.

The common sample retained 58.7% to 91.1% of sampled students by system (46,442 of 64,168 overall, 72.4%). Retention was not random with respect to achievement: included students outscored excluded students by 18 to 47 points on mathematics achievement, and the proportion of girls was 4 to 17 percentage points higher among the included. The estimates therefore pertain to students enrolled concurrently in all four science subjects within self-selected systems, and are not generalized to all Grade 8 students. Participant flow by system appears in Table 2 and in full in Section S1 of the Supplementary Materials.

### 3.3. Measures

*Subject confidence.* Confidence was assessed by eight statements per subject on a four-point agreement scale from 1 (*agree a lot*) to 4 (*disagree a lot*), four positively worded (e.g., “I usually do well in biology”) and four negatively worded (e.g., “Biology is not one of my strengths”). Analyses employed the released Rasch partial-credit scale scores (BSBGSCB, BSBGSCE, BSBGSCC, BSBGSCP, BSBGSCM), scaled by the assessment agency to an international centerpoint of 10 and a standard deviation of 2 within cycle. TIMSS designates these scales as measures of confidence, whereas the theoretical framework invoked here derives from research on academic self-concept, and the two labels are not interchangeable. The item content corresponds closely to the competence component of academic self-concept as conventionally operationalized: the items ask students to appraise how well they do in the subject relative to an implicit standard, which is the content of the competence dimension in the Marsh–Shavelson tradition (Marsh, 1990; Marsh & Shavelson, 1985), and they contain neither the affective items that distinguish the affect dimension nor the task-specific mastery judgments that distinguish self-efficacy. That distinction is not merely terminological. Marsh et al. (2019) document how readily self-concept, self-efficacy, and self-confidence are conflated under interchangeable labels, and Pēčiuliauskienė (2020) shows that the TIMSS confidence scales carry variance attributable to both self-concept and self-efficacy. The scales are accordingly treated as indicators of the competence component that the constructs share, the term *confidence* is used throughout to preserve the distinction, and comparisons with the subject-specific self-concept literature are drawn at that level rather than at the level of the broader construct. The released scores were retained as the primary specification for three reasons: they are the scores on which the assessment agency’s own reporting and the existing comparative literature rest; they use all eight items and are produced by a common Rasch model within cycle, so departures from that model are diagnosable rather than concealed; and recomputing the outcome from a subset of items changes the construct as well as its reliability. The wording failures documented in Section 4.2 are therefore addressed by reporting a positively-worded-items-only specification and a three-system exclusion as sensitivity analyses rather than by replacing the primary outcome.

*Achievement.* Five plausible values were employed for overall mathematics (BSMMAT01–05) and for each science content domain: biology (BSSBIO01–05), chemistry (BSSCHE01–05), physics (BSSPHY01–05), and earth science (BSSEAR01–05). The *m*th plausible value of each scale was paired with the *m*th plausible value of every other scale, following the recommended procedure. Overall mathematics achievement is used as the anchor although TIMSS Grade 8 mathematics is itself reported in four content domains (Number, Algebra, Geometry and Measurement, Data and Probability). The hypotheses concern mathematics as a curricular domain rather than any particular mathematical competence, and the comparison subjects are defined at the level of school subjects, so an aggregate anchor is the quantity the theory refers to. Because aggregating across mathematics domains could nonetheless conceal heterogeneity of the kind that motivates disaggregating science, each mathematics content domain (BSMNUM, BSMALG, BSMGEO, BSMDAT, five plausible values each) was substituted for the overall score in a sensitivity analysis reported in Section 4.9 and Section S9.

*Covariates.* Student sex (ITSEX; 1 = *girl*, 2 = *boy*) and home educational resources (BSBGHER, the released scale) were entered in all models.

### 3.4. Statistical Analyses

The analysis proceeded in three stages: estimation within each education system, propagation of the uncertainty attaching to the achievement scaling and the sampling design, and synthesis of the system-specific estimates.

*Estimation within systems.* A single regression model was fitted separately within each of the 11 systems. Confidence in each of the five subjects was regressed simultaneously on achievement in all five subjects, together with student sex and home educational resources, with all 25 achievement coefficients freely estimated:

(1) $$y_{\mathrm{ik}}=\beta_{0k}+\sum_{j=1}^{5}\beta_{\mathrm{jk}}\;x_{\mathrm{ij}}+\gamma_{k}^{⊤}c_{i}+\varepsilon_{ik}$$

where *y_ik_* is the standardized confidence of student *i* in subject *k*, *x_ij_* is standardized achievement in domain *j*, and **c***_i_* is the covariate vector. Entering all five achievement scores at once is what renders the coefficient of interest interpretable: the association between mathematics achievement and biology confidence is estimated with achievement in biology, chemistry, physics, and earth science held constant, and therefore reflects what is the residual component of mathematics achievement that remains after conditioning on the four science achievement indicators, rather than general academic strength. The four coefficients relating mathematics achievement to the four science confidence scales constitute the primary estimand.

Two contrasts among those four coefficients were specified in advance. The first compared the two subjects of lower anticipated quantitative demand with the two of higher demand, and the second compared the extremes:

*δ*_1_ = 1/2[(*β*_mat, bio_ + *β*_mat, ear_) − (*β*_mat, che_ + *β*_mat, phy_)],  *δ*_2_ = *β*_mat, bio_ − *β*_mat, phy_ (2)

A third set of contrasts, used to test Hypothesis 2, took the difference between each forward association (mathematics achievement with science confidence) and its reverse counterpart (science achievement with mathematics confidence).

Estimation was by weighted least squares using the total student weight (TOTWGT) without normalization. Weighted means and standard deviations of all variables were recomputed within every system, plausible value, and replicate, so that uncertainty in the standardizers was carried through rather than fixed.

*Nested conditioning sets.* Because the mathematics coefficient in Equation (1) is a partial coefficient estimated under pronounced collinearity, its relation to the corresponding marginal association is not self-evident. Three further specifications were therefore estimated for each science confidence outcome, identically weighted and with identical uncertainty propagation: the zero-order association with mathematics achievement; the association conditioning on achievement in the corresponding science subject alone; and the association conditioning on overall science achievement (BSSSCI01–05). These are reported alongside the full specification in Table 3 so that the effect of each conditioning step is visible rather than inferred.

*Propagation of uncertainty.* Each model was refitted twice over. It was fitted once for each of the five plausible values representing achievement, and once for each replicate weight representing the complex sampling design. Sampling variance was obtained by the full TIMSS jackknife repeated replication procedure, in which both complementary replicates were formed for each sampling zone—one doubling the weight of the first primary sampling unit and suppressing the second, the other reversing that assignment—and the sum of squared deviations from the full-sample estimate was multiplied by one half, the factor appropriate when both complementary replicates are used (Fishbein et al., 2025). The number of zones was determined empirically for each system and ranged from 67 to 125. Sampling and imputation variance were combined by Rubin’s (1987) rules across the five plausible values.

This procedure was applied not only to individual coefficients but to every contrast. Each contrast was computed inside every replicate and every plausible value before its variance was calculated, which preserves the covariance among coefficients estimated from the same students. Comparing separately reported estimates and their standard errors would ignore that covariance and misstate the precision of the difference. Cases with missing covariates were excluded from the corresponding equation, and the exclusion was applied identically within every replicate.

### 3.5. The Placebo Anchor

Hypothesis 3 asserts that the within-science cross-paths change when mathematics achievement is introduced. A comparison of the unanchored and anchored specifications is only informative if the change cannot be produced by the arithmetic of adding a correlated predictor, and it is worth establishing precisely what that arithmetic implies. Let *y* be a confidence outcome and *X* the design matrix containing the four science achievement scores and the covariates. Let *P* = *Xa* + *e* be a candidate anchor whose fitted component lies in the column space of *X* and whose residual *e* is orthogonal to *X* under the estimation weights. Because the column spaces of [*X*, *P*] and [*X*, *e*] coincide, the coefficients on *X* in the anchored model equal the unanchored coefficients minus *a* times the coefficient on *e*. If *e* is unrelated to *y*, that coefficient has expectation zero, and the science coefficients are unchanged in expectation. The mechanical attenuation produced by adding a covariance-matched but conditionally uninformative predictor is therefore exactly zero rather than merely small, and any systematic change observed on adding mathematics achievement is attributable to its conditional association with the outcome. It also follows that adding a correlated predictor may reduce, increase, or reverse an existing coefficient depending on the covariance structure; the earlier claim that such addition necessarily reduces existing coefficients was incorrect and has been withdrawn.

The placebo anchor operationalizes that argument and is retained as a numerical validation of it. The mathematics plausible value was regressed on the four science plausible values, and its residual was replaced by random noise made orthogonal to the science achievement scores under the estimation weights and rescaled to the residual standard deviation. The resulting variable reproduces the sample covariance of mathematics achievement with each science predictor exactly rather than in expectation; this was verified numerically in every system, with a maximum relative discrepancy of 2.7 × 10^−13^, at the limit of floating-point precision. Its fitted component still carries a marginal association with confidence through the science achievement scores, so the comparison concerns conditional rather than marginal relations. The placebo was constructed separately within each system and each plausible value. The number of draws was increased from 20 to 200 in revision (seed 20260809) and a convergence check is reported: the mean absolute discrepancy between the placebo coefficient and the unanchored coefficient across the 12 cross-paths and 11 systems fell from 0.0021 at 5 draws to 0.00098 at 20, 0.00055 at 50, 0.00035 at 100, and 0.00033 at 200, and the median Monte Carlo standard error of the placebo mean fell from 0.0019 to 0.00036 over the same range. At 200 draws, the Monte Carlo component is roughly one twentieth of the smallest sampling standard error in Section 4.6 and is therefore negligible; at 20 draws it was already an order of magnitude below it, so the original choice did not affect any conclusion. The convergence of the placebo mean on the unanchored coefficient is the numerical counterpart of the algebraic result above. Because the mechanical baseline is zero, Hypothesis 3 is tested by the direct anchored-minus-unanchored difference rather than by an anchored-minus-placebo difference. The direct comparison is formed inside every jackknife replicate and every plausible value before its variance is computed, which retains the covariance between the two specifications and yields standard errors roughly five times smaller than those obtained by differencing independently estimated quantities. Three specifications are reported for every within-science association in Section 4.6: unanchored, anchored, and placebo.

### 3.6. Synthesis Across Systems

System-specific estimates were combined by random-effects meta-analysis. Between-system variance was estimated by restricted maximum likelihood, which is less biased than method-of-moments alternatives when heterogeneity is substantial (Viechtbauer, 2005), and each system was weighted by the inverse of its sampling variance plus that between-system component. Inference used the Hartung–Knapp–Sidik–Jonkman adjustment (Hartung & Knapp, 2001; Sidik & Jonkman, 2002), which rescales the standard error by the observed dispersion of the system estimates around the pooled value and refers the result to a *t* distribution on *k* − 1 degrees of freedom. The adjustment is preferable to normal-theory intervals when the number of studies is small and heterogeneity is appreciable, as it is here.

Three quantities are reported for every pooled estimate. The confidence interval describes the precision of the average association. The between-system standard deviation and *I*^2^ describe how far systems differ from one another. The prediction interval describes the range within which the association would be expected to fall in a further system exchangeable with those analysed, and is calculated from the between-system variance and the standard error of the mean on *k* − 2 degrees of freedom. Prediction intervals are reported throughout because, where heterogeneity is substantial, the confidence interval for the mean does not describe the range of system-level values (IntHout et al., 2016). Leave-one-system-out estimates are reported as influence diagnostics.

Hypothesis 4 concerns the ordering of four coefficients rather than the heterogeneity of each considered separately, and its four graded claims require different treatment. Claims (a) and (b), concerning the sign of the biology–physics contrast and the position of biology, were evaluated by synthesizing the prespecified ordering contrasts, which retain the within-system covariance among coefficients, by tabulating the observed configuration in every system, and by computing the probability of each ordering event under each system’s estimated sampling distribution. Because observed ranks ignore sampling error and adjacent coefficients may exchange rank by chance, each system’s four coefficients were drawn 20,000 times from independent normal distributions with the estimated means and standard errors, and the proportion of draws satisfying each event was recorded. Claim (c), the complete rank order, was evaluated the same way; claim (d), homogeneity of magnitudes, by the heterogeneity statistics of the meta-analysis.

*Multiplicity.* The confirmatory family was fixed in advance and comprises twelve estimates: the four mathematics-anchored associations, the two prespecified ordering contrasts, the four dependent asymmetry contrasts, and the two physics–chemistry attenuation comparisons. These address four logically separate hypotheses, and no single correction across all twelve would be interpretable; adjustment is therefore applied within each hypothesis family, which is the level at which the inferential claims are made. Holm adjustment is applied within two families: the four mathematics-anchored coefficients bearing on Hypothesis 1, and the two physics–chemistry attenuation comparisons bearing on Hypothesis 3. Benjamini–Hochberg values are reported alongside for the former. The asymmetry contrasts bearing on Hypothesis 2 and the ordering contrasts bearing on Hypothesis 4 are not adjusted, because in each case the inferential claim rests on a single prespecified quantity rather than on the most extreme of several. Both unadjusted and Holm-adjusted *p* values are reported in the Abstract, Results, and Discussion wherever an adjusted family is involved, and the tables retain the unadjusted values so that readers can apply an alternative convention. All estimates outside the confirmatory family are descriptive, are labelled as such, and are not accompanied by adjusted values.

### 3.7. Sensitivity Analyses

Seven were conducted and all are reported. Five were prespecified: outcome-specific against common samples (Section 4.8); unanchored against anchored specifications (Section 4.6); confidence recomputed from the four positively worded items only (Section 4.8); exclusion of the three systems in which negatively worded items failed to reverse (Section 4.8); and re-estimation including France and Portugal (Section 4.8 and Section S8). Two were added in revision: the nested sequence of conditioning sets reported in Table 3, and the substitution of each mathematics content domain for the overall mathematics anchor reported in Section 4.9. Collinearity diagnostics were computed on the first plausible value and verified against the remaining four, and the proportion of mathematics achievement variance surviving the conditioning is reported in Section 4.1.

## 4. Results

### 4.1. Coverage, Selection, and Collinearity

All derived context scales and achievement scales required by the design were present under the anticipated designations. Coverage of the subject-specific blocks and analytic sample sizes appear in Table 2. Retention into the common sample ranged from 58.7% (Finland) to 91.1% (Hungary), and included students differed systematically from excluded students on achievement and sex, as reported above and in Section S1.

Condition numbers for the five-predictor achievement block ranged from 4.0 to 7.7 and the maximum variance inflation factor was 9.7. These values indicate that the matrix is invertible, but 9.7 approaches conventional warning thresholds and the diagnostics are heuristics rather than criteria. Because the substantive conclusions depend on the sign and relative magnitude of partial coefficients rather than on prediction, a more directly interpretable quantity is the share of mathematics achievement that survives the conditioning. Regressing mathematics achievement on the four science content domains within each system returned *R*^2^ values of 0.478 to 0.704 (median 0.616), so the residual on which the mathematics coefficient is estimated retains 29.6% to 52.2% of the variance of mathematics achievement (median 38.4%). The conditioning is therefore substantial but does not reduce the predictor to a narrow remainder. Weighted correlations among the four science content domains ranged from 0.67 to 0.90 (median 0.85) and between mathematics and each science domain from 0.59 to 0.81 (median 0.74). Coefficient stability across plausible values, across the three alternative parameterizations of the achievement block reported in Table 3, and across the alternative mathematics anchors reported in Section 4.9 is reported in preference to reliance on the collinearity diagnostics themselves.

### 4.2. Measurement Diagnostics

Reliability of the eight-item confidence scales varied markedly across systems. In Azerbaijan, Morocco, and Uzbekistan the negatively worded items did not reverse: their correlations with the positively worded anchor item were positive, and coefficient alpha computed on all eight items ranged from 0.58 to 0.72, against 0.84 to 0.94 for the four positively worded items alone. In the remaining eight systems all four negatively worded items reversed as intended. Full diagnostics by system and subject appear in Section S2. This pattern is consistent with careless or acquiescent responding of the kind documented in TIMSS motivation scales by Michaelides (2019), and it constitutes a direct threat to cross-system comparability of the released scores. It is addressed analytically below rather than only in the limitations.

### 4.3. Duplicated Response Blocks in France and Portugal

**Figure 1:**
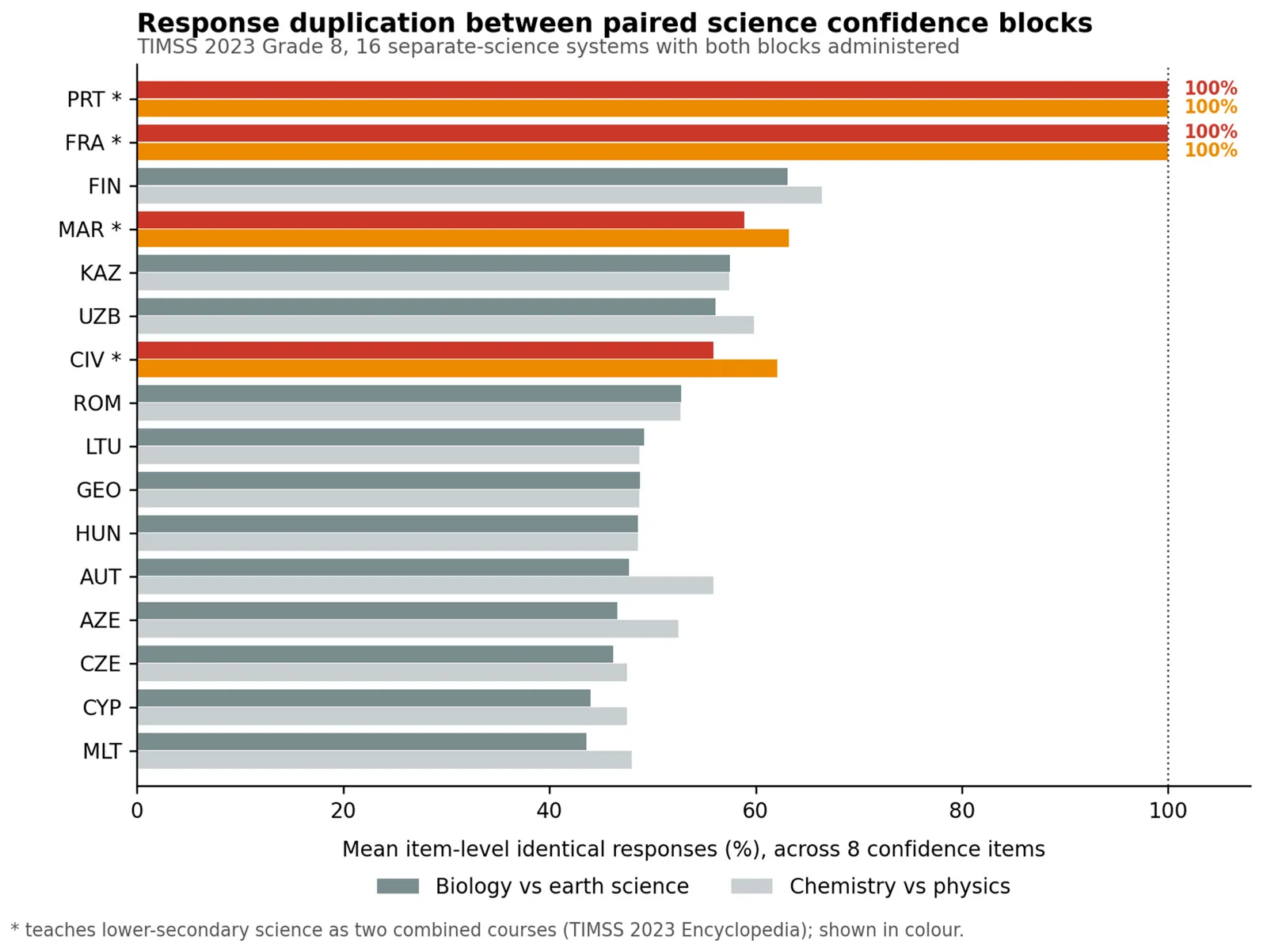
Response Duplication Between Paired Science Confidence Blocks. *Note.* Bars denote mean unweighted item-level identical responses across the eight confidence items; pair-specific *n* ranged from 581 to 7722 and is reported in full in Section S3. Systems marked with an asterisk deliver lower-secondary science as two combined courses and are additionally distinguished by colour (biology versus earth science in red, chemistry versus physics in orange); all other systems are shown in grey (biology versus earth science in dark grey, chemistry versus physics in light grey). France and Portugal returned complete duplication; Morocco and Côte d’Ivoire, with equivalent curricular organization, did not. AUT = Austria; AZE = Azerbaijan; CIV = Côte d’Ivoire; CYP = Cyprus; CZE = Czechia; FIN = Finland; FRA = France; GEO = Georgia; HUN = Hungary; KAZ = Kazakhstan; LTU = Lithuania; MAR = Morocco; MLT = Malta; PRT = Portugal; ROM = Romania; UZB = Uzbekistan.

Responses to the paired blocks were identical in France and Portugal: mean agreement across the eight confidence items reached 100.0% with *r* = 1.000 for biology against earth science and for chemistry against physics in both systems (pair-specific *n* = 3913 to 5134, unweighted). Across the remaining 14 systems the same comparisons ranged from 43.6% to 66.9% (Figure 1). The configuration did not track curricular organization: Morocco and Côte d’Ivoire deliver lower-secondary science as the same two combined courses and returned agreement of 58.9% and 55.9% for biology against earth science. Two independently completed eight-item blocks cannot agree on every case. The duplication rule was specified as mean item-level agreement exceeding 95% and was applied before any structural model was estimated. The source of the duplication has not been established with the data producer, and the exclusion is therefore reported as provisional, with sensitivity analyses including both systems in Section S8.

### 4.4. Mathematics-Anchored Partial Associations

The reported coefficients are partial regression coefficients estimated under pronounced collinearity, and their interpretation depends on what conditioning removes. Table 3 therefore reports the association of mathematics achievement with each confidence scale under four nested specifications, from the zero-order association to the model conditioning on all four science content domains. The zero-order associations are positive for every subject (β = 0.122 to 0.232), as would be expected given that all achievement and confidence measures share general academic standing. Conditioning on science achievement reverses the sign for biology and, to a smaller extent, for earth science, while leaving the chemistry and physics associations positive and reduced in magnitude. The reversal is not an artefact of the particular conditioning set: it appears when only the corresponding subject’s achievement is controlled (β = −0.165), when overall science achievement is controlled (β = −0.231), and when the four content domains are entered jointly (β = −0.158). The sign of the biology coefficient is thus a property of the conditional estimand rather than of the marginal relation, and it is reported throughout as an association with the residual variation in mathematics achievement that remains after science achievement is held constant.

**Figure 2:**
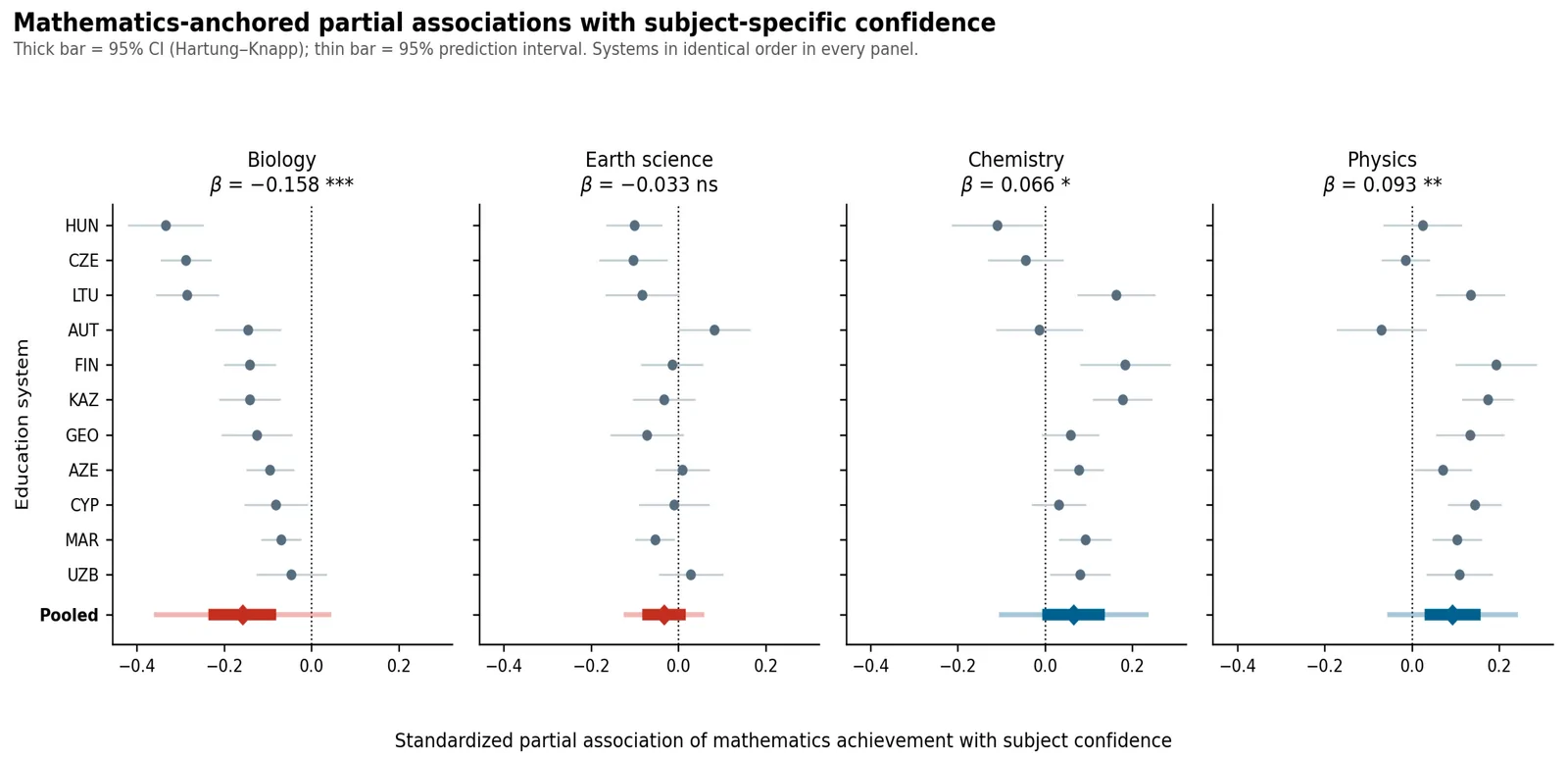
Mathematics-Anchored Partial Associations With Subject-Specific Confidence. *Note.* Points denote system-specific standardized partial associations with 95% confidence intervals. Diamonds denote pooled estimates; thick bars denote 95% Hartung–Knapp confidence intervals and thin bars 95% prediction intervals. Pooled estimates are shown in red where the pooled association is negative (biology, earth science) and in blue where it is positive (chemistry, physics). Systems appear in identical order in every panel, sorted by the biology estimate. AUT = Austria; AZE = Azerbaijan; CYP = Cyprus; CZE = Czechia; FIN = Finland; GEO = Georgia; HUN = Hungary; KAZ = Kazakhstan; LTU = Lithuania; MAR = Morocco; UZB = Uzbekistan. *k* = 11. ns = not significant; * *p* < 0.05; ** *p* < 0.01; *** *p* < 0.001.

Table 4 and Figure 2 report the four associations of primary interest under the full specification; the complete 5 × 5 coefficient matrix from which they are drawn appears in Table 5. The association was negative for biology confidence (β = −0.158, 95% CI [−0.224, −0.093], *p* < 0.001) and positive for physics confidence (β = 0.093, 95% CI [0.041, 0.146], *p* = 0.003). The association for chemistry confidence was positive and reached conventional significance (β = 0.066, 95% CI [0.006, 0.125], *p* = 0.035), and the association for earth science confidence was not distinguishable from zero (β = −0.033, 95% CI [−0.070, 0.005], *p* = 0.084). Because the confirmatory family contains four such estimates, Holm-adjusted *p* values are also reported: biology *p* = 0.001 and physics *p* = 0.009 survive adjustment, whereas chemistry does not (*p* = 0.072; Benjamini–Hochberg *p* = 0.048) and earth science does not (*p* = 0.086). Each coefficient describes the association with the residual component of mathematics achievement after the four science domains are partialled out; that residual retains 29.6% to 52.2% of the variance of mathematics achievement by system (median 38.4%), so the conditioning does not reduce the predictor to a negligible remainder.

Between-system heterogeneity was substantial for all four associations (*I*^2^ = 55.9% to 87.3%; $\hat{\tau }$= 0.037 to 0.085). The 95% prediction intervals consequently included zero in every case: [−0.362, 0.045] for biology, [−0.125, 0.060] for earth science, [−0.106, 0.237] for chemistry, and [−0.057, 0.243] for physics. Leave-one-system-out estimates ranged from −0.169 to −0.142 for biology and from 0.084 to 0.107 for physics, indicating that no single system drives either estimate. Heterogeneity of this magnitude is treated here as a finding in its own right rather than as a caveat: the pooled values describe average associations among the included systems, and the prediction intervals indicate that the association in a further exchangeable system could take either sign for every one of the four subjects.

Hypothesis 1 was therefore supported for biology and physics and not supported for earth science; the chemistry association was positive as predicted but proved specification-dependent, losing significance both under adjustment for multiplicity and under two of the three sensitivity specifications reported in Section 4.8, and it is characterized as inconclusive rather than as a positive association. The prespecified ordering contrasts were both negative and significant (δ_1_ = −0.172, 95% CI [−0.235, −0.109], *p* < 0.001; δ_2_ = −0.249, 95% CI [−0.319, −0.179], *p* < 0.001). Of the six confirmatory estimates—the four mathematics-anchored associations and the two ordering contrasts—δ_2_ was the only one whose prediction interval excluded zero ([−0.455, −0.042]); the interval for δ_1_ was [−0.358, 0.014]. Several coefficients outside the confirmatory family, including the matching associations and the physics-with-biology cross-path, also returned prediction intervals excluding zero (Section S4).

### 4.5. Symmetry of the Associations

Hypothesis 2 was not supported. None of the four dependent asymmetry contrasts differed from zero: α_bio_ = −0.045, 95% CI [−0.102, 0.013], *p* = 0.117; α_ear_ = 0.019, 95% CI [−0.045, 0.083], *p* = 0.519; α_che_ = −0.028, 95% CI [−0.132, 0.076], *p* = 0.559; α_phy_ = 0.022, 95% CI [−0.070, 0.114], *p* = 0.608. Forward and reverse associations were of comparable magnitude for every subject, and the asymmetry reported by Jansen et al. (2015) was not reproduced. This is a failure to detect asymmetry and not a demonstration of symmetry. The intervals exclude absolute asymmetries larger than approximately 0.10 for biology, 0.08 for earth science, 0.13 for chemistry, and 0.11 for physics; asymmetries smaller than those bounds are compatible with these data, and with *k* = 11 systems the design cannot exclude them. Two further considerations qualify the comparison independently of precision: the compared coefficients have different outcome variables of unequal reliability (Section S2), and they derive from two regression equations estimated on a single cross-sectional occasion, so neither notation nor covariate adjustment establishes a direction of influence.

### 4.6. Within-Science Cross-Paths and the Placebo Comparison

The remaining ten cross-paths are exploratory and behaved heterogeneously (Table 6; system-level values in Section S10). Three were amplified rather than attenuated, one reversed sign (biology confidence on chemistry achievement, from −0.017 to 0.039), and the three paths terminating in biology confidence changed most. This pattern demonstrates empirically what Section 3.5 establishes algebraically: introducing a correlated predictor does not attenuate existing coefficients as a matter of arithmetic, but can reduce, increase, or reverse them according to the covariance structure.

Under the unanchored specification, the within-science configuration corresponded to that reported by Guo et al. (2017) in sign for the two paths they emphasized: physics achievement with biology confidence was negative (β = −0.115) and chemistry achievement with physics confidence was positive (β = 0.104). A formal replication criterion was not prespecified, and the complete twelve-path pattern with uncertainty is reported in Table 6 and Section S4 rather than characterized by selected coefficients.

### 4.7. Cross-System Ordering

Hypothesis 4 requires separation into the distinct claims that the term *invariance* conflates: invariance of the sign of a single coefficient, invariance of a specific pairwise contrast, invariance of the complete rank order of four coefficients, and equality of their magnitudes. The evidence differs sharply across them. Biology returned the most negative association in 11 of 11 systems, and the biology–physics contrast was ordered in the predicted direction in 11 of 11. The complete four-subject rank order was reproduced in 7 of 11 systems: Austria, Hungary, Kazakhstan, and Lithuania departed from it (Table 7). Magnitudes were not invariant at all, as the heterogeneity statistics in Section 4.4 establish. Observed ranks do not account for sampling error, and two adjacent coefficients may exchange rank by chance alone. Each system’s four coefficients were therefore resampled 200,000 times from their joint sampling distribution and the probability of each ordering event computed (Table 8). Because the four coefficients are estimated from the same students in the same multivariate regression, their sampling errors are correlated, and the resampling uses the full 4 × 4 sampling covariance matrix recovered from the replicate-level estimates rather than four independent normal distributions; the median absolute correlation among the four coefficients is 0.46 and the maximum is 0.91, so the independence assumption is not tenable. The probability that the complete four-subject order is reproduced exceeds 0.50 in only 5 of 11 systems and falls to 0.000 in Austria, whereas the probability that biology is the most negative of the four ranges from 0.660 to 1.000 and the probability that biology falls below physics ranges from 0.824 to 1.000, reaching 1.000 in 10 of 11 systems. Accounting for the covariance raises most of the complete-order probabilities and raises the lower bound on the biology–physics comparison, so the restricted conclusion that follows is if anything better supported than under the earlier calculation. Hypothesis 4 is therefore supported in the restricted sense that the sign of the biology–physics contrast is stable across every system examined, and is not supported for the complete ordering of the four subjects or for the magnitudes. This restricted conclusion is the one carried into the Discussion; the earlier framing, which treated ordering invariance as a single hypothesis, overstated the regularity that these data support.

### 4.8. Sensitivity Analyses

The five sensitivity analyses specified in Section 3.7 are reported in full here and in Sections S5 and S7–S10. *Measurement specifications.* The biology association was robust: β = −0.158 in the primary specification, −0.192 excluding the three systems with wording failures (*p* = 0.001), and −0.178 using the four positively worded items only (*p* < 0.001). The physics association was similarly stable (0.093, 0.090, 0.072; all *p* < 0.05). The chemistry association was not: 0.066 in the primary specification but 0.056 (*p* = 0.200) excluding the three systems and 0.033 (*p* = 0.348) using positively worded items only. The earth science association remained small and of inconsistent significance (−0.033, *p* = 0.084; −0.044, *p* = 0.085; −0.053, *p* = 0.031). *Analytic sample.* Outcome-specific and common-sample estimates were near-identical for every subject (biology −0.158 against −0.159; earth science −0.032 against −0.035; chemistry 0.065 against 0.065; physics 0.093 against 0.092), so the choice of sample does not bear on any conclusion. *Anchor specification.* The unanchored-against-anchored comparison is reported in Section 4.6 and Table 6. *France and Portugal.* Re-estimating all models with both systems retained (*k* = 13) left every conclusion intact and strengthened three of the four estimates: biology −0.163 (95% CI [−0.218, −0.109]), earth science −0.054 (*p* = 0.028), chemistry 0.074 (*p* = 0.008), physics 0.098 (*p* < 0.001). Both systems returned identical biology and earth science coefficients (−0.202 in France, −0.186 in Portugal), which is a direct consequence of the block duplication and is itself further evidence of it. No conclusion in this article depends on the exclusion of these two systems. Full results appear in Section S8.

### 4.9. Mathematics Content Domains as Alternative Anchors

The biology association is insensitive to the choice (Table 9): it ranges from −0.130 to −0.142 across the four domains against −0.158 for the overall score, and is significant at *p* ≤ 0.001 in every case. The physics association is positive under all four, though it weakens when data and probability is the anchor (0.048, *p* = 0.063). The chemistry association is the least stable: it ranges from 0.008 with the data and probability anchor to 0.080 with the algebra anchor, and reaches conventional significance with only three of the four. Entering all four mathematics domains simultaneously reproduces this contrast, with chemistry confidence associated positively with algebra (0.119, *p* = 0.010) and negatively with data and probability (−0.115, *p* = 0.012) while no mathematics domain is individually associated with biology confidence once the others are held constant (all *p* ≥ 0.150). Two conclusions follow. The negative biology association, which carries most of the article’s substantive weight, does not depend on which aspect of mathematics achievement serves as the anchor. The chemistry association does, which is consistent with its instability across the measurement specifications reported in Section 4.8 and reinforces its characterization as inconclusive. Aggregating across mathematics domains is therefore defensible for the estimates on which the conclusions rest, and an overall anchor is retained as primary because the hypotheses concern mathematics as a curricular domain rather than any particular mathematical competence; the disaggregated results are reported so that readers can see where that aggregation matters.

## 5. Discussion

The main goal of the present study was to estimate the partial association of mathematics achievement with confidence in four science subjects across the education systems administering subject-specific questionnaires in TIMSS 2023. A secondary goal concerned attenuation of within-science cross-paths under the anchor, and a third the extent of between-system heterogeneity.

The first finding was that subject-specific science confidence scales do not relate uniformly to mathematics achievement. Biology confidence was negatively associated and physics confidence positively associated, and the difference between them was the single estimate in the study whose prediction interval excluded zero. The magnitude of the biology association, β = −0.158, is comparable to cross-domain associations reported between mathematics and native-language domains in the classical I/E literature (Marsh, 1986; Möller et al., 2020). This finding must be stated together with the conditioning that produces it. The zero-order association between mathematics achievement and biology confidence is positive (β = 0.122), as it is for all four subjects, and becomes negative only once science achievement is held constant, whether by the corresponding subject alone, by overall science achievement, or by the four content domains jointly (Table 3). The sign of the biology coefficient is thus a property of the conditional estimand and not of the marginal relation, and it is reported as such. The corollary for measurement practice is unaffected: an aggregate that averages biology and physics confidence combines constituents whose conditional associations with mathematics achievement are of opposing sign, and the chemistry estimate is too specification-dependent to be counted alongside them.

That corollary requires care in its formulation. The present study did not analyze an aggregate score, did not compare hierarchical against domain-specific measurement models, and did not examine the predictive consequences of aggregation. Differential associations of this kind do not preclude a meaningful higher-order science factor, and the evidence that such a factor is defensible is substantial (Arens et al., 2021; Schroeders & Jansen, 2022). The warranted conclusion is narrower: aggregation across science subjects can obscure associations of opposing sign with mathematics achievement, and the interpretation of an aggregate should not be assumed to transfer to its constituents. A related distinction concerns the construct itself. TIMSS designates the outcome scales as measures of confidence, whereas the theoretical framework invoked here derives from research on academic self-concept. The item content corresponds closely to the competence component of academic self-concept as conventionally operationalized, and the two studies have long been intertwined; but the labels are not interchangeable, and Marsh et al. (2019) document how readily self-concept, self-efficacy, and self-confidence are conflated under interchangeable names. Pēčiuliauskienė (2020) shows that the TIMSS confidence scales carry both self-concept and self-efficacy variance. The comparisons drawn here with the subject-specific self-concept literature are therefore made at the level of the competence component the instruments share, and results should not be read as bearing on the affective or the efficacy components that the TIMSS scales do not isolate.

The second finding concerns the characterizations of biology self-concept summarized in the Introduction. Biology confidence was the most negatively associated of the four subjects in every system examined, which is consistent with the direction reported by Guo et al. (2017) and not with the mathematics-like characterization of Arens et al. (2018). Several qualifications are essential, and they are of different kinds. A negative partial association with mathematics achievement is not evidence that a subject occupies a verbal terminus; no verbal anchor was estimated, and none is available in TIMSS at Grade 8. The divergent prior characterizations arose in different national samples, instruments, and tracks, and the substantial heterogeneity documented here is itself consistent with moderation of the kind that would reconcile them rather than adjudicate between them. Most importantly, dimensional comparison is not the only process that could generate the result. The residual component of mathematics achievement on which the coefficient is estimated may also carry quantitative reasoning that the science content-domain scores measure less well, differential curricular exposure, selection into courses, tracking, and the imperfect correspondence between TIMSS content-domain proficiency and achievement in separately taught school subjects. The design cannot separate these from dimensional comparison, and the finding is reported as a conditional association whose interpretation remains open rather than as a demonstration of a comparison process.

The third and fourth findings concern the two hypotheses that were framed as comparisons rather than as single coefficients, and the evidence divides them. Hypothesis 3 received support once the change in each cross-path was formed inside every replicate and plausible value rather than compared across separately estimated quantities. Both prespecified physics–chemistry paths were attenuated by the introduction of the mathematics anchor: physics achievement with chemistry confidence moved from 0.013 to 0.001 (Δ = −0.014, unadjusted *p* = 0.040, Holm-adjusted *p* = 0.040) and chemistry achievement with physics confidence from 0.104 to 0.081 (Δ = −0.027, unadjusted *p* = 0.012, Holm-adjusted *p* = 0.024). Both remain below 0.05 after adjustment within the two-comparison Hypothesis 3 family. The magnitudes are small, and the appropriate reading is not that the association reported by Guo et al. (2017) is explained by shared quantitative content but that a measurable part of it is shared with mathematics achievement. The remaining ten cross-paths behaved heterogeneously: three were amplified, one reversed in sign, and the paths terminating in biology confidence changed most. That pattern is itself informative, because it demonstrates directly what the placebo analysis establishes analytically, namely that conditioning on a correlated predictor does not produce attenuation as a matter of arithmetic. Hypothesis 2 was not supported, and the appropriate conclusion is that the study did not detect asymmetry rather than that forward and reverse associations are equivalent. The confidence intervals for the four asymmetry contrasts exclude absolute differences larger than approximately 0.08 to 0.13; asymmetries smaller than that remain entirely compatible with these data, and with *k* = 11 systems the design has limited capacity to exclude them. The same reasoning applies to every null reported here. Two further considerations qualify the comparison independently of power: the forward and reverse coefficients have different outcome variables whose reliabilities differ, as Table S2 documents, and the two directions are separate regression equations estimated on one cross-sectional measurement occasion, so neither notation nor adjustment establishes a direction of influence.

The fourth finding is that between-system heterogeneity is better regarded as a substantive result than as a qualification of the pooled estimates. *I*^2^ ranged from 56% to 87% and the 95% prediction interval included zero for all four individual associations, so the study establishes average associations among the included systems without establishing that an association of the same magnitude, or necessarily of the same sign, would be observed elsewhere. Two classes of explanation deserve separate treatment. Substantive moderators plausibly include the degree to which science subjects are differentiated in the curriculum, the presence and timing of tracking, the mathematical content of science courses as taught, subject enrolment patterns, and system-level differences in perceived subject similarity (Helm et al., 2016); the correspondence between TIMSS content domains and the subjects in which students are actually enrolled is a further candidate, since that correspondence is itself a curricular property that varies across systems. Measurement explanations are equally admissible: the wording failures documented in Azerbaijan, Morocco, and Uzbekistan indicate that part of the observed variation may reflect non-equivalence of the released scores rather than variation in dimensional comparison processes, and the excess heterogeneity is not confined to those systems. Formal meta-regression is not advisable with *k* = 11, and none is reported; the distinction between the two classes of explanation is therefore drawn conceptually and left for designs with more systems or with direct measures of the proposed moderators. What the heterogeneity does establish is a boundary condition on dimensional comparison theory as applied to within-science comparisons: whatever process generates the biology–physics divergence operates with a magnitude that is system-dependent, and a theory that predicts only the direction of that divergence is consistent with the data while a theory that predicts its size is not.

### 5.1. Implications

Four follow. First, reports of aggregate science motivation in international assessments warrant qualification for systems delivering discrete science subjects, because the constituent scales relate to mathematics achievement with associations of opposing sign. Second, the interpretation of subject-specific confidence scales requires attention to wording effects, which in three of eleven systems were severe enough to halve the reliability of the released eight-item scores; the recent literature on inconsistent responding in international assessments (Konstantinidou & Michaelides, 2025; Michaelides et al., 2026; Steinmann et al., 2024) indicates that this is a general rather than an incidental problem, and that it is systematically related to achievement and sex, which are precisely the variables such comparisons condition on. Third, the estimation of anchored associations rather than their inference from within-domain patterns is a tractable design extensible to any assessment furnishing matched achievement and self-concept measures at subject level; whether such associations correspond to positions on a continuum is a further question requiring a verbal anchor and a direct measure of appraised similarity. Fourth, and most consequential for practice, the magnitude of between-system heterogeneity means that a pooled international estimate is a poor guide to any particular system: system-level estimates, not averages, are the appropriate input to system-level decisions.

### 5.2. Limitations

The design is cross-sectional and all estimates are conditional associations; no temporal or causal ordering is identified, and the covariate adjustment and directional notation do not establish direction. Appraised similarity was not measured. No verbal anchor was available, so no claim is advanced regarding position between termini. Confidence entered as released Rasch scale scores rather than as latent variables, so outcome measurement error and wording-method variance were not modelled; the consequence for standardized coefficients depends on the measurement model and is not a simple attenuation. Three restrictions on generalization require emphasis together rather than separately. First, the 11 systems are neither a random sample of TIMSS systems nor a random sample of education systems: they are the systems that elected to administer the separate-science questionnaire and that met the inclusion criteria. Second, within those systems the common sample comprises students concurrently enrolled in all four science subjects, retention ranged from 58.7% to 91.1%, and included students outscored excluded students by 18 to 47 points on mathematics achievement with 4 to 17 percentage points more girls. Third, the prediction intervals reported throughout should therefore be read as applying, at most, to a further education system exchangeable with those included, and not to TIMSS systems or to education systems in general. Statements about what would be expected in “a further education system” have been qualified accordingly throughout the manuscript. Achievement predictors are content-domain scores from a matrix-sampled science assessment rather than course-specific performance measures, and they are strongly intercorrelated; the mathematics coefficient accordingly describes the residual component of mathematics achievement and not mathematics-specific competence. The exclusion of France and Portugal rests on statistical implausibility rather than on verification with the data producer, and the principal conclusions are unchanged when both systems are retained (Section 4.8). Finally, the study estimates 25 coefficients per system together with multiple contrasts; the confirmatory family is restricted to the four mathematics-anchored associations, the two ordering contrasts, the four asymmetry contrasts, and the two physics–chemistry attenuation comparisons, Holm-adjusted *p* values are reported for that family, and all remaining estimates are descriptive.

## Figures and Tables

**Table 1:** Divergent Characterizations of Biology Self-Concept in Prior Work.

Characterization	Evidentiary Basis	Source	Sample
Mathematics-like	Contrast from native-language achievement	Arens et al. (2018)	Germany, *N* = 1230, two ability tracks
Neither terminus	Weak or null cross-paths in both directions	Jansen et al. (2015); Marsh et al. (2015b)	Germany, *N* = 20,050
Verbal-like	Contrast with physics	Guo et al. (2017)	TIMSS 2007, four countries, *N* = 18,047

*Note.* The three characterizations derive from different samples, instruments, tracks, and model specifications and are not necessarily contradictory.

**Table 2:** Participant Flow, Coverage, and Collinearity by Education System.

System	Sampled *N*	Bio %	Earth %	Chem %	Phys %	Math %	Common *N*	Retention %	Cond. No.	Max VIF
Austria	4639	92.7	90.1	90.4	85.8	95.4	3497	75.4	6.6	7.5
Azerbaijan	6174	79.8	78.9	75.6	76.5	85.6	4054	65.7	6.0	6.3
Cyprus	4200	87.1	77.9	83.7	82.0	91.5	2806	66.8	6.7	7.4
Czechia	8110	95.0	93.3	93.4	93.5	97.0	6766	83.4	6.8	7.9
Finland	5880	79.9	85.4	78.6	73.5	93.8	3454	58.7	6.9	7.8
Georgia	4872	86.2	85.2	85.0	83.7	91.5	3396	69.7	5.9	6.2
Hungary	5426	96.7	96.4	96.1	96.2	97.7	4945	91.1	7.1	8.3
Kazakhstan	6362	87.9	84.8	84.1	85.0	93.5	4422	69.5	6.1	6.4
Lithuania	5421	91.3	91.0	89.7	89.6	93.8	4534	83.6	7.7	9.7
Morocco	8745	87.0	82.7	80.0	84.0	91.3	5695	65.1	4.0	3.2
Uzbekistan	4339	85.3	82.4	84.3	85.1	93.0	2873	66.2	5.4	5.0
Total	64,168						46,442	72.4		

*Note.* Sampled *N* is the unweighted count of students in the released student file. Percentages are unweighted valid responses on each subject’s confidence scale. Common *N* is the unweighted count with valid data on all five confidence scales, both covariates, and the design variables; it is the sample used for contrasts spanning equations. Outcome-specific samples are larger and are reported in Section S1. Condition numbers and variance inflation factors pertain to the five-predictor achievement block computed on the first plausible value and verified against the remaining four. Six further systems were excluded: Côte d’Ivoire (no science content-domain plausible values), Sweden (earth science block not administered), Romania and Malta (coverage below 50% on at least one subject), France and Portugal (duplicated response blocks).

**Table 3:** Mathematics–Confidence Associations Under Four Nested Specifications.

Confidence	Model 0: Zero-Order	Model 1: + Own-Subject Science Achievement	Model 2: + Overall Science Achievement	Model 3: + Four Science Content Domains
Biology	0.122 (0.016) ***	−0.165 (0.031) ***	−0.231 (0.037) ***	−0.158 (0.029) ***
Earth science	0.169 (0.015) ***	−0.016 (0.019)	−0.069 (0.018) **	−0.032 (0.017)
Chemistry	0.214 (0.024) ***	0.074 (0.028) *	0.073 (0.031) *	0.065 (0.027) *
Physics	0.232 (0.013) ***	0.106 (0.017) ***	0.095 (0.027) **	0.093 (0.024) **

*Note.* Pooled standardized coefficients with standard errors in parentheses; *k* = 11 education systems, random-effects meta-analysis with the Hartung–Knapp adjustment. Model 0 is the weighted bivariate association with no covariates. Models 1–3 include student sex and home educational resources. Model 3 is the specification used throughout the remainder of the article. * *p* < 0.05. ** *p* < 0.01. *** *p* < 0.001.

**Table 4:** Pooled Mathematics-Anchored Partial Associations With Subject Confidence.

Outcome	β	*SE*	*t*(10)	*p*	95% CI	95% PI	$\hat{\tau }$	*I*^2^ (%)	LOO Range
Biology	−0.158	0.029	−5.40	<0.001	[−0.224, −0.093]	[−0.362, 0.045]	0.085	87.3	[−0.169, −0.142]
Earth science	−0.033	0.017	−1.92	0.084	[−0.070, 0.005]	[−0.125, 0.060]	0.037	55.9	[−0.042, −0.025]
Chemistry	0.066	0.027	2.44	0.035	[0.006, 0.125]	[−0.106, 0.237]	0.071	75.7	[0.054, 0.080]
Physics	0.093	0.024	3.95	0.003	[0.041, 0.146]	[−0.057, 0.243]	0.062	76.6	[0.084, 0.107]
Ordering contrasts									
δ_1_ (life vs. physical)	−0.172	0.028	−6.09	<0.001	[−0.235, −0.109]	[−0.358, 0.014]	0.077	82.9	
δ_2_ (biology − physics)	−0.249	0.031	−7.90	<0.001	[−0.319, −0.179]	[−0.455, −0.042]	0.086	80.0	

*Note. k* = 11 education systems. Estimates are standardized partial regression coefficients conditional on achievement in all four science content domains, student sex, and home educational resources, obtained on outcome-specific samples. Inference is by restricted maximum likelihood random-effects meta-analysis with the Hartung–Knapp adjustment. PI = prediction interval; LOO = leave-one-system-out range of the pooled estimate. Ordering contrasts are computed on the common sample and defined in Equation (2).

**Table 5:** Pooled Standardized Coefficient Matrix.

Confidence	Bio Ach.	Earth Ach.	Chem Ach.	Phys Ach.	Math Ach.
Biology	0.363 (0.036) ***	0.057 (0.025) *	0.041 (0.023)	−0.079 (0.024) **	−0.159 (0.030) ***
Earth science	0.161 (0.032) ***	0.117 (0.043) *	0.001 (0.026)	−0.052 (0.037)	−0.035 (0.017)
Chemistry	0.048 (0.030)	0.003 (0.033)	0.156 (0.032) ***	0.003 (0.030)	0.065 (0.028) *
Physics	−0.018 (0.036)	−0.019 (0.031)	0.078 (0.034) *	0.100 (0.020) ***	0.093 (0.024) **
Mathematics	−0.119 (0.038) *	−0.066 (0.023) *	0.082 (0.024) **	0.071 (0.026) *	0.420 (0.028) ***

*Note.* Standard errors in parentheses. Matching associations appear on the diagonal. Estimates derive from the common sample (*N* = 46,442) so that the matrix is internally consistent; outcome-specific estimates for the mathematics column appear in Table 4 and differ negligibly. Complete results with confidence intervals, heterogeneity statistics, and outcome-specific sample sizes appear in Section S4. * *p* < 0.05. ** *p* < 0.01. *** *p* < 0.001.

**Table 6:** Within-Science Cross-Paths Under Three Anchor Specifications.

Confidence	Achievement	Unanchored	Anchored	Placebo	Δ (Anchored − Unanchored)	SE	*p*
Biology	Earth science	0.033	0.057	0.032	+0.023	0.006	0.005
Biology	Chemistry	−0.017	0.039	−0.004	+0.051	0.012	0.002
Biology	Physics	−0.115	−0.079	−0.114	+0.033	0.005	<0.001
Earth science	Biology	0.155	0.158	0.155	+0.003	0.001	0.075
Earth science	Chemistry	−0.006	0.001	−0.006	+0.009	0.005	0.113
Earth science	Physics	−0.059	−0.053	−0.060	+0.008	0.003	0.042
Chemistry	Biology	0.050	0.045	0.050	−0.004	0.002	0.118
Chemistry	Earth science	0.011	0.004	0.011	−0.009	0.004	0.077
Chemistry	Physics	0.013	0.001	0.013	−0.014	0.006	0.040
Physics	Biology	−0.009	−0.019	−0.009	−0.006	0.002	0.027
Physics	Earth science	−0.009	−0.019	−0.010	−0.012	0.003	0.005
Physics	Chemistry	0.104	0.081	0.104	−0.027	0.009	0.012

*Note.* Pooled standardized coefficients, *k* = 11 education systems, common sample. *Unanchored* conditions on the four science content domains and the covariates; *anchored* adds mathematics achievement; *placebo* adds the covariance-matched placebo anchor averaged over *R* = 200 draws (seed 20260809). Δ is the anchored-minus-unanchored difference formed inside every jackknife replicate and every plausible value before its variance was computed, which retains the covariance between the two specifications; it is a difference in signed coefficients. The placebo column is reported as validation rather than as a comparison baseline: its expectation equals the unanchored coefficient (Section 3.5), and the observed mean absolute discrepancy was 0.00033. The two rows constituting the confirmatory test of Hypothesis 3 are chemistry confidence with physics achievement and physics confidence with chemistry achievement; the remaining ten comparisons are exploratory.

**Table 7:** Rank Order of Mathematics-Anchored Associations Within Each System.

System	Rank Order	Biology	Earth Science	Chemistry	Physics
Austria	bio < phy < che < ear	−0.136	0.079	−0.024	−0.082
Azerbaijan	bio < ear < che < phy	−0.101	−0.001	0.074	0.083
Cyprus	bio < ear < che < phy	−0.061	−0.002	0.038	0.138
Czechia	bio < ear < che < phy	−0.289	−0.098	−0.051	−0.013
Finland	bio < ear < che < phy	−0.137	−0.014	0.174	0.184
Georgia	bio < ear < che < phy	−0.120	−0.083	0.048	0.134
Hungary	bio < che < ear < phy	−0.333	−0.103	−0.108	0.028
Kazakhstan	bio < ear < phy < che	−0.152	−0.039	0.188	0.182
Lithuania	bio < ear < phy < che	−0.296	−0.084	0.172	0.134
Morocco	bio < ear < che < phy	−0.066	−0.059	0.099	0.106
Uzbekistan	bio < ear < che < phy	−0.059	0.035	0.081	0.106

*Note.* Biology returned the most negative association in 11 of 11 systems. The complete four-subject order was reproduced in 7 of 11.

**Table 8:** Probability of Ordering Events Within Each Education System.

System	Observed Rank Order	*P*(Complete Order) Joint	*P*(Complete Order) Independent	*P*(Biology Most Negative)	*P*(Biology < Physics)
Austria	bio < phy < che < ear	0.000	0.001	0.819	0.824
Azerbaijan	bio < ear < che < phy	0.542	0.524	0.998	1.000
Cyprus	bio < ear < che < phy	0.685	0.566	0.854	1.000
Czechia	bio < ear < che < phy	0.468	0.524	1.000	1.000
Finland	bio < ear < che < phy	0.669	0.552	1.000	1.000
Georgia	bio < ear < che < phy	0.780	0.650	0.795	1.000
Hungary	bio < che < ear < phy	0.440	0.442	1.000	1.000
Kazakhstan	bio < ear < phy < che	0.405	0.441	1.000	1.000
Lithuania	bio < ear < phy < che	0.225	0.283	1.000	1.000
Morocco	bio < ear < che < phy	0.413	0.324	0.660	1.000
Uzbekistan	bio < ear < che < phy	0.738	0.419	0.998	1.000

*Note.* Each system’s four mathematics-anchored coefficients were drawn 200,000 times from a multivariate normal distribution with the estimated means and the full 4 × 4 sampling covariance matrix, and the proportion of draws satisfying each ordering event is reported. The covariance was formed from the replicate-level estimates: the jackknife covariance was computed within each plausible value and combined across plausible values by the matrix form of Rubin’s rules. Because the four coefficients are estimated from the same students in the same multivariate regression, their sampling errors are correlated; the column headed “independent” reproduces the earlier calculation, which treated them as independent, so that the consequence of the correction is visible. The complete order is biology < earth science < chemistry < physics. Probabilities are conditional on the estimated sampling covariance and do not incorporate between-system heterogeneity.

**Table 9:** Pooled Associations With Subject Confidence Under Alternative Mathematics Anchors.

Mathematics Anchor	Biology	Earth Science	Chemistry	Physics
Overall mathematics	−0.158 ***	−0.032	0.065 *	0.093 **
Number	−0.142 ***	−0.018	0.065 *	0.096 ***
Algebra	−0.139 ***	−0.034 *	0.080 *	0.085 **
Geometry and measurement	−0.130 **	−0.051 **	0.041	0.082 **
Data and probability	−0.138 ***	−0.021	0.008	0.048

*Note. k* = 11 education systems. Each row substitutes a different mathematics scale for overall mathematics achievement; all models condition on achievement in the four science content domains, student sex, and home educational resources. Standardized coefficients from random-effects meta-analysis with the Hartung–Knapp adjustment. * *p* < 0.05. ** *p* < 0.01. *** *p* < 0.001.

## Data Availability

This study analyzed publicly available data from the TIMSS 2023 database (https://timss2023.org/data/ (accessed on 2 October 2026).

## Supplementary Materials

The following supporting information can be downloaded at: https://www.mdpi.com/article/10.3390/bs16101924/s1, Table S1: Participant flow by education system; Table S2: Reliability and item polarity by system and subject; Table S3: Item-level agreement between paired confidence blocks; Table S4: All 25 pooled standardized associations with confidence intervals, prediction intervals, and heterogeneity; Table S5: Mathematics-anchored association under alternative specifications; Table S6: Ordering and asymmetry contrasts within each education system; Table S7a: Mathematics coefficient under four nested conditioning sets, by system; Table S7b: Variance of mathematics achievement surviving the conditioning; Table S8: Mathematics-anchored associations with france and portugal retained (k = 13); Table S9: Associations under alternative mathematics anchors, by system; Table S10: Anchored minus unanchored cross-path differences by system; Table S11: Correlations among the four mathematics-anchored coefficients within each education system.

## References

[B1-behavsci-16-01924] Arens A. K., Becker M., Möller J. (2018). The internal/external frame of reference model: Extension to five school subjects and invariance across German secondary school ability tracks. Learning and Individual Differences.

[B2-behavsci-16-01924] Arens A. K., Jansen M., Preckel F., Schmidt I., Brunner M. (2021). The structure of academic self-concept: A methodological review and empirical illustration of central models. Review of Educational Research.

[B3-behavsci-16-01924] Chiu M.-S. (2008). Achievements and self-concepts in a comparison of math and science: Exploring the internal/external frame of reference model across 28 countries. Educational Research and Evaluation.

[B4-behavsci-16-01924] Chiu M.-S. (2012). The internal/external frame of reference model, big-fish-little-pond effect, and combined model for mathematics and science. Journal of Educational Psychology.

[B5-behavsci-16-01924] Ding Y., Yang Hansen K., Klapp A. (2023). Testing measurement invariance of mathematics self-concept and self-efficacy in PISA using MGCFA and the alignment method. European Journal of Psychology of Education.

[B6-behavsci-16-01924] Fishbein B., Taneva M., Kowolik K. (2025). TIMSS 2023 user guide for the international database.

[B7-behavsci-16-01924] Guo J., Marsh H. W., Parker P. D., Dicke T. (2018). Cross-cultural generalizability of social and dimensional comparison effects on reading, math, and science self-concepts for primary school students using the combined PIRLS and TIMSS data. Learning and Instruction.

[B8-behavsci-16-01924] Guo J., Marsh H. W., Parker P. D., Morin A. J. S., Dicke T. (2017). Extending expectancy-value theory predictions of achievement and aspirations in science: Dimensional comparison processes and expectancy-by-value interactions. Learning and Instruction.

[B9-behavsci-16-01924] Hartung J., Knapp G. (2001). On tests of the overall treatment effect in meta-analysis with normally distributed responses. Statistics in Medicine.

[B10-behavsci-16-01924] He J., Barrera-Pedemonte F., Buchholz J. (2019). Cross-cultural comparability of noncognitive constructs in TIMSS and PISA. Assessment in Education: Principles, Policy & Practice.

[B11-behavsci-16-01924] Helm F., Mueller-Kalthoff H., Nagy N., Möller J. (2016). Dimensional comparison theory: Perceived subject similarity impacts on students’ self-concepts. AERA Open.

[B12-behavsci-16-01924] IntHout J., Ioannidis J. P. A., Rovers M. M., Goeman J. J. (2016). Plea for routinely presenting prediction intervals in meta-analysis. BMJ Open.

[B13-behavsci-16-01924] Jansen M., Schroeders U., Lüdtke O. (2014). Academic self-concept in science: Multidimensionality, relations to achievement measures, and gender differences. Learning and Individual Differences.

[B14-behavsci-16-01924] Jansen M., Schroeders U., Lüdtke O., Marsh H. W. (2015). Contrast and assimilation effects of dimensional comparisons in five subjects: An extension of the I/E model. Journal of Educational Psychology.

[B15-behavsci-16-01924] Jansen M., Schroeders U., Lüdtke O., Marsh H. W. (2019). The dimensional structure of students’ self-concept and interest in science depends on course composition. Learning and Instruction.

[B16-behavsci-16-01924] Konstantinidou E., Michaelides M. P. (2025). Assessment mode and inconsistent responding on a mixed-worded scale: Evidence from TIMSS 2019 across grades and countries. Frontiers in Education.

[B17-behavsci-16-01924] Marsh H. W. (1986). Verbal and math self-concepts: An internal/external frame of reference model. American Educational Research Journal.

[B18-behavsci-16-01924] Marsh H. W. (1990). The structure of academic self-concept: The Marsh/Shavelson model. Journal of Educational Psychology.

[B19-behavsci-16-01924] Marsh H. W., Abduljabbar A. S., Parker P. D., Morin A. J. S., Abdelfattah F., Nagengast B., Möller J., Abu-Hilal M. M. (2015a). The internal/external frame of reference model of self-concept and achievement relations: Age-cohort and cross-cultural differences. American Educational Research Journal.

[B20-behavsci-16-01924] Marsh H. W., Lüdtke O., Nagengast B., Trautwein U., Abduljabbar A. S., Abdelfattah F., Jansen M. (2015b). Dimensional comparison theory: Paradoxical relations between self-beliefs and achievements in multiple domains. Learning and Instruction.

[B21-behavsci-16-01924] Marsh H. W., Pekrun R., Parker P. D., Murayama K., Guo J., Dicke T., Arens A. K. (2019). The murky distinction between self-concept and self-efficacy: Beware of lurking jingle-jangle fallacies. Journal of Educational Psychology.

[B22-behavsci-16-01924] Marsh H. W., Shavelson R. (1985). Self-concept: Its multifaceted, hierarchical structure. Educational Psychologist.

[B23-behavsci-16-01924] Michaelides M. P. (2019). Negative keying effects in the factor structure of TIMSS 2011 motivation scales and associations with reading achievement. Applied Measurement in Education.

[B24-behavsci-16-01924] Michaelides M. P., Konstantinidou E., Ivanova M., Steinmann I. (2026). Inconsistent responding on a mixed-worded scale in the PISA 2022 questionnaire: Prevalence and predictors across seven countries. Journal of Psychoeducational Assessment.

[B25-behavsci-16-01924] Möller J. (2024). Ten years of dimensional comparison theory: On the development of a theory from educational psychology. Educational Psychology Review.

[B26-behavsci-16-01924] Möller J., Marsh H. W. (2013). Dimensional comparison theory. Psychological Review.

[B27-behavsci-16-01924] Möller J., Zitzmann S., Helm F., Machts N., Wolff F. (2020). A meta-analysis of relations between achievement and self-concept. Review of Educational Research.

[B28-behavsci-16-01924] Ofqual (2020). GCE subject level conditions and requirements for science (biology, chemistry, physics) and certificate requirements (2021).

[B29-behavsci-16-01924] Palmgren E., Kokkonen T., Bruun J. (2025). Roles of mathematics in physics education: A systematic review. Physical Review Physics Education Research.

[B30-behavsci-16-01924] Pēčiuliauskienė P. (2020). School students’ self-confidence in science and intrinsic motivation for learning science: Self-concept and self-efficacy approach. Pedagogika.

[B31-behavsci-16-01924] Rubin D. B. (1987). Multiple imputation for nonresponse in surveys.

[B32-behavsci-16-01924] Rutkowski D., Rutkowski L., Hastedt D. (2026). Student achievement and self-confidence in mathematics and science: Evidence from TIMSS 2023. Large-scale Assessments in Education.

[B33-behavsci-16-01924] Saß S., Kampa N. (2019). Self-concept profiles in lower secondary level—An explanation for gender differences in science course selection?. Frontiers in Psychology.

[B34-behavsci-16-01924] Schroeders U., Jansen M. (2022). Science self-concept—More than the sum of its parts?. The Journal of Experimental Education.

[B35-behavsci-16-01924] Sidik K., Jonkman J. N. (2002). A simple confidence interval for meta-analysis. Statistics in Medicine.

[B36-behavsci-16-01924] Steinmann I., Chen J., Braeken J. (2024). Who responds inconsistently to mixed-worded scales? Differences by achievement, age group, and gender. Assessment in Education: Principles, Policy & Practice.

[B37-behavsci-16-01924] Viechtbauer W. (2005). Bias and efficiency of meta-analytic variance estimators in the random-effects model. Journal of Educational and Behavioral Statistics.

[B38-behavsci-16-01924] Wurster S. (2022). Measurement invariance of non-cognitive measures in TIMSS across countries and across time. Studies in Educational Evaluation.

[B39-behavsci-16-01924] Ye S., Elmgren M., Jacobsson M., Ho F. M. (2024). How much is just maths? Investigating problem solving in chemical kinetics at the interface of chemistry and mathematics through the development of an extended mathematical modelling cycle. Chemistry Education Research and Practice.

