# Supplementary material for: Mathematics Partial Associations with Subject-Specific Science Confidence: Evidence from 11 Education Systems in TIMSS 2023
Source: Behav Sci (Basel). 2026 Oct 17;16(10):1924. doi: 10.3390/bs16101924 (PMC13701797; doi:10.3390/bs16101924)
Supplement: Supplementary file 1 [file behavsci-16-01924-s001.zip › behavsci-4540729-supplementary.pdf]

Supplementary Material

Mathematics-Anchored Partial Associations With Subject-Specific Science Confidence: Evidence From 11 Education Systems in TIMSS 2023

Georgios D. Sideridis and Mohammed Alghamdi

This document contains supplementary tables and methodological detail referenced in the main manuscript. Table and section numbering is prefixed with S. Sections S7–S11 were added in revision in response to reviewer comments.

Section S1. Participant Flow and Selection Into the Analytic Samples

Table S1 reports, for each system, the sampled count, the count with valid data on each confidence scale, the count with valid covariates, and the common-sample count requiring all five confidence scales together with both covariates and the design variables. The final columns report the difference between included and excluded students on mathematics achievement, biology achievement, the percentage of girls, and home educational resources.

Table S1

Participant Flow by Education System

System	Sampled	Bio	Earth	Chem	Phys	Math	Covariates	Common N	Retention %	Δ math	Δ bio	Δ % girls	Δ resources
Austria	4639	4301	4180	4195	3982	4426	4593	3497	75.4	22.1	21.6	4.2	−0.10
Azerbaijan	6174	4927	4873	4669	4726	5282	5926	4054	65.7	46.6	39.9	13.5	0.39
Cyprus	4200	3659	3270	3517	3446	3844	4121	2806	66.8	21.6	30.0	17.4	0.29
Czechia	8110	7705	7568	7576	7586	7867	8048	6766	83.4	34.8	37.2	16.2	0.29
Finland	5880	4696	5022	4622	4320	5516	5752	3454	58.7	39.8	49.0	10.9	0.28
Georgia	4872	4199	4149	4143	4080	4458	4750	3396	69.7	45.4	50.6	13.4	0.34
Hungary	5426	5249	5228	5213	5218	5301	5353	4945	91.1	37.6	31.7	9.5	0.51
Kazakhstan	6362	5594	5396	5348	5410	5949	6250	4422	69.5	44.4	54.9	7.2	0.36
Lithuania	5421	4949	4932	4865	4859	5086	5312	4534	83.6	28.5	32.4	8.3	0.14
Morocco	8745	7609	7228	6998	7343	7982	8448	5695	65.1	18.2	12.0	7.7	−0.08
Uzbekistan	4339	3699	3576	3656	3691	4036	4306	2873	66.2	24.3	26.7	7.8	0.26

Note. Counts are unweighted. Δ columns give the mean difference (included minus excluded). Retention into the common sample ranged from 58.7% to 91.1%; included students consistently outperformed excluded students on achievement, and the proportion of girls was higher among the included in every system. The estimates therefore pertain to students concurrently enrolled in all four science subjects.

Section S2. Reliability and Wording Diagnostics

Each confidence block comprises eight items, four positively and four negatively worded. Item polarity was assessed by correlating every item with the positively worded anchor item ("I usually do well in [subject]"). In a well-behaved block all four negatively worded items correlate negatively with the anchor. Table S2 reports the number that did so, coefficient alpha computed on all eight items after reverse coding, and coefficient alpha computed on the four positively worded items alone.

Table S2

Reliability and Item Polarity by System and Subject

System	Subject	n	Negative items reversing	Mean r with anchor	α (8 items)	α (4 positive)
Austria	Biology	4094	4	−0.293	0.832	0.866
Austria	Earth science	3942	4	−0.296	0.840	0.888
Austria	Chemistry	3999	4	−0.312	0.844	0.899
Austria	Physics	3770	4	−0.283	0.837	0.899
Austria	Mathematics	4223	4	−0.483	0.896	0.877
Azerbaijan	Biology	4351	0	0.077	0.689	0.896
Azerbaijan	Earth science	4296	0	0.119	0.666	0.911
Azerbaijan	Chemistry	4108	0	0.129	0.694	0.938
Azerbaijan	Physics	4188	0	0.142	0.667	0.927
Azerbaijan	Mathematics	4673	4	−0.105	0.796	0.899
Cyprus	Biology	3397	4	−0.220	0.838	0.900
Cyprus	Earth science	3036	4	−0.093	0.792	0.912
Cyprus	Chemistry	3241	4	−0.143	0.819	0.910
Cyprus	Physics	3197	4	−0.135	0.816	0.922
Cyprus	Mathematics	3622	4	−0.372	0.880	0.901
Czechia	Biology	7304	4	−0.468	0.878	0.850
Czechia	Earth science	7220	4	−0.507	0.893	0.879
Czechia	Chemistry	7264	4	−0.487	0.888	0.877
Czechia	Physics	7265	4	−0.485	0.891	0.892
Czechia	Mathematics	7598	4	−0.590	0.911	0.872
Finland	Biology	4514	4	−0.231	0.821	0.895
Finland	Earth science	4844	4	−0.244	0.829	0.900
Finland	Chemistry	4449	4	−0.360	0.875	0.924
Finland	Physics	4138	4	−0.342	0.873	0.930
Finland	Mathematics	5325	4	−0.466	0.906	0.920
Georgia	Biology	3853	4	−0.239	0.824	0.878
Georgia	Earth science	3856	4	−0.170	0.804	0.891
Georgia	Chemistry	3778	4	−0.210	0.825	0.906
Georgia	Physics	3712	4	−0.231	0.836	0.915
Georgia	Mathematics	4106	4	−0.356	0.860	0.883
Hungary	Biology	4981	4	−0.409	0.877	0.885
Hungary	Earth science	4962	4	−0.390	0.872	0.893
Hungary	Chemistry	4922	4	−0.416	0.880	0.904
Hungary	Physics	4927	4	−0.376	0.872	0.903
Hungary	Mathematics	5019	4	−0.524	0.908	0.891
Kazakhstan	Biology	5270	4	−0.186	0.814	0.868
Kazakhstan	Earth science	5066	4	−0.154	0.799	0.882
Kazakhstan	Chemistry	5040	4	−0.187	0.819	0.897
Kazakhstan	Physics	5090	4	−0.176	0.816	0.899
Kazakhstan	Mathematics	5623	4	−0.342	0.865	0.883
Lithuania	Biology	4654	4	−0.147	0.778	0.860
Lithuania	Earth science	4614	4	−0.132	0.763	0.873
Lithuania	Chemistry	4568	4	−0.182	0.796	0.889
Lithuania	Physics	4558	4	−0.158	0.799	0.907
Lithuania	Mathematics	4801	4	−0.370	0.857	0.876
Morocco	Biology	6639	0	0.079	0.656	0.872
Morocco	Earth science	6268	0	0.134	0.590	0.868
Morocco	Chemistry	6086	0	0.131	0.588	0.869
Morocco	Physics	6325	0	0.145	0.580	0.874
Morocco	Mathematics	6975	3	0.009	0.693	0.843
Uzbekistan	Biology	3410	2	−0.004	0.723	0.857
Uzbekistan	Earth science	3292	0	0.059	0.672	0.869
Uzbekistan	Chemistry	3333	0	0.063	0.692	0.893
Uzbekistan	Physics	3385	0	0.109	0.654	0.888
Uzbekistan	Mathematics	3696	4	−0.079	0.770	0.871

Note. *In Azerbaijan, Morocco, and Uzbekistan the negatively worded items did not reverse: their correlations with the anchor item were positive, and alpha computed on all eight items fell to .58–.72 against .84–.94 for the positively worded items alone. 13 of 55 system-by-subject blocks were affected, all confined to those three systems. The released Rasch scale scores incorporate all eight items and therefore inherit this contamination.*

Section S3. Duplicated Response Blocks

The duplication rule was specified as mean item-level agreement exceeding 95% across the eight confidence items of a paired block, and was applied before any structural model was estimated. Agreement statistics are unweighted. The substantive re-estimation including France and Portugal, which Reviewer 3 correctly noted was referenced but not reported, now appears in Section S8.

Table S3

Item-Level Agreement Between Paired Confidence Blocks

System	Pair	n	% identical	Mean r
FRA	Chemistry-Physics	3916	100.0	1.000
PRT	Biology-Earth sci	5094	100.0	1.000
FRA	Biology-Earth sci	3913	100.0	1.000
PRT	Chemistry-Physics	5134	100.0	1.000
SWE	Chemistry-Physics	2743	66.9	0.639
FIN	Chemistry-Physics	3670	66.4	0.680
MAR	Chemistry-Physics	6197	63.2	0.601
FIN	Biology-Earth sci	4267	63.1	0.592
CIV	Chemistry-Physics	7722	62.1	0.540
UZB	Chemistry-Physics	3293	59.8	0.496
MAR	Biology-Earth sci	6469	58.9	0.561
KAZ	Biology-Earth sci	5000	57.5	0.454
KAZ	Chemistry-Physics	4930	57.4	0.459
UZB	Biology-Earth sci	3183	56.1	0.439
CIV	Biology-Earth sci	6912	55.9	0.433
AUT	Chemistry-Physics	3775	55.9	0.491
ROM	Biology-Earth sci	1283	52.8	0.477
ROM	Chemistry-Physics	2806	52.7	0.486
AZE	Chemistry-Physics	4204	52.5	0.461
LTU	Biology-Earth sci	4745	49.2	0.367
GEO	Biology-Earth sci	3780	48.8	0.370
GEO	Chemistry-Physics	3759	48.7	0.402
LTU	Chemistry-Physics	4652	48.7	0.389
HUN	Chemistry-Physics	5059	48.6	0.378
HUN	Biology-Earth sci	5087	48.6	0.307
MLT	Chemistry-Physics	670	48.0	0.359
AUT	Biology-Earth sci	3991	47.7	0.323
CYP	Chemistry-Physics	3223	47.5	0.397
CZE	Chemistry-Physics	7232	47.5	0.344
AZE	Biology-Earth sci	4391	46.6	0.350
CZE	Biology-Earth sci	7293	46.2	0.264
CYP	Biology-Earth sci	3075	44.0	0.330
MLT	Biology-Earth sci	581	43.6	0.272

Note. *France and Portugal returned complete duplication on both pairs. Morocco and Côte d'Ivoire deliver lower-secondary science as the same two combined courses and did not. The source of the duplication has not been verified with the data producer, and the exclusion is provisional; Section S8 shows that no conclusion depends on it.*

Section S4. Complete Coefficient Matrix With Uncertainty

Table S4

All 25 Pooled Standardized Associations With Confidence Intervals, Prediction Intervals, and Heterogeneity

Confidence	Achievement	β	SE	p	CI lo	CI hi	PI lo	PI hi	τ	I²
Biology	Biology	0.363	0.036	0.000	0.283	0.443	0.180	0.546	0.073	43.6
Biology	Chemistry	0.041	0.023	0.109	−0.011	0.093	−0.021	0.103	0.015	0.0
Biology	Earth science	0.057	0.025	0.046	0.001	0.112	−0.044	0.157	0.037	17.0
Biology	Mathematics	−0.159	0.030	0.000	−0.226	−0.093	−0.367	0.048	0.087	86.5
Biology	Physics	−0.079	0.024	0.009	−0.133	−0.025	−0.134	−0.024	0.000	14.4
Chemistry	Biology	0.048	0.030	0.136	−0.018	0.115	−0.041	0.138	0.026	19.3
Chemistry	Chemistry	0.156	0.032	0.001	0.084	0.228	−0.027	0.339	0.074	57.1
Chemistry	Earth science	0.003	0.033	0.921	−0.070	0.077	−0.173	0.179	0.070	52.6
Chemistry	Mathematics	0.065	0.028	0.042	0.003	0.128	−0.114	0.244	0.074	75.2
Chemistry	Physics	0.003	0.030	0.920	−0.063	0.069	−0.123	0.129	0.047	25.0
Earth science	Biology	0.161	0.032	0.000	0.091	0.232	0.090	0.233	0.000	0.0
Earth science	Chemistry	0.001	0.026	0.980	−0.057	0.059	−0.058	0.060	0.000	0.0
Earth science	Earth science	0.117	0.043	0.022	0.021	0.214	−0.140	0.374	0.105	63.0
Earth science	Mathematics	−0.035	0.017	0.063	−0.073	0.002	−0.122	0.051	0.034	50.4
Earth science	Physics	−0.052	0.037	0.197	−0.135	0.032	−0.231	0.128	0.070	47.1
Mathematics	Biology	−0.119	0.038	0.011	−0.204	−0.033	−0.349	0.111	0.094	66.7
Mathematics	Chemistry	0.082	0.024	0.007	0.028	0.135	0.006	0.158	0.023	28.6
Mathematics	Earth science	−0.066	0.023	0.015	−0.117	−0.016	−0.117	−0.015	0.000	0.0
Mathematics	Mathematics	0.420	0.028	0.000	0.358	0.483	0.224	0.617	0.082	86.9
Mathematics	Physics	0.071	0.026	0.022	0.012	0.129	−0.044	0.186	0.043	30.5
Physics	Biology	−0.018	0.036	0.627	−0.099	0.062	−0.186	0.150	0.065	40.2
Physics	Chemistry	0.078	0.034	0.046	0.002	0.154	−0.075	0.231	0.059	52.3
Physics	Earth science	−0.019	0.031	0.550	−0.089	0.050	−0.198	0.159	0.072	58.8
Physics	Mathematics	0.093	0.024	0.003	0.039	0.146	−0.059	0.244	0.063	73.6
Physics	Physics	0.100	0.020	0.001	0.055	0.145	0.054	0.146	0.000	0.0

Note. k = 11. Estimates derive from the common sample. Inference by restricted maximum likelihood with the Hartung–Knapp adjustment on 10 degrees of freedom. PI = prediction interval.

Section S5. Sensitivity Analyses

Table S5

Mathematics-Anchored Association Under Alternative Specifications

Confidence	Primary β	Primary p	Excluding 3 systems β	Excluding 3 systems p	Positive items only β	Positive items only p
Biology	−0.159	0.000	−0.192	0.001	−0.178	0.000
Earth science	−0.035	0.063	−0.044	0.085	−0.053	0.031
Chemistry	0.065	0.042	0.056	0.200	0.033	0.348
Physics	0.093	0.003	0.090	0.034	0.072	0.037

Note. "Excluding 3 systems" omits Azerbaijan, Morocco, and Uzbekistan, in which negatively worded items failed to reverse. "Positive items only" recomputes each outcome as the mean of the four positively worded items, reversed so that higher values denote greater confidence. The biology and physics associations were stable across all three specifications; the chemistry association was not, and it is characterized as inconclusive in the revised manuscript.

Section S6. Prespecified Contrasts by System

Table S6

Ordering and Asymmetry Contrasts Within Each Education System

System	α bio	α che	α ear	α phy	δ_1	δ_2
Austria	0.117	−0.285	0.252	−0.186	0.025	−0.054
Azerbaijan	−0.091	−0.004	0.028	0.088	−0.129	−0.184
Cyprus	0.092	−0.025	0.049	0.023	−0.119	−0.199
Czechia	−0.005	−0.176	−0.122	−0.213	−0.162	−0.276
Finland	0.038	−0.037	0.049	0.188	−0.254	−0.322
Georgia	−0.141	0.020	0.021	0.037	−0.192	−0.254
Hungary	−0.030	−0.322	−0.170	−0.002	−0.178	−0.361
Kazakhstan	−0.059	0.196	0.051	0.058	−0.280	−0.333
Lithuania	−0.226	0.074	−0.040	0.254	−0.343	−0.430
Morocco	−0.059	0.103	0.041	−0.008	−0.165	−0.172
Uzbekistan	−0.090	0.017	0.190	0.065	−0.105	−0.164

Note. *δ_1 and δ_2 are defined in Equation 2; α are the dependent asymmetry contrasts. Each was evaluated within every jackknife replicate and every plausible value before combination, so that the covariance among coefficients estimated from the same students is retained.*

Section S7. Nested Conditioning Sequence by Education System

Added in revision in response to Reviewer 1 (comment 1) and Reviewer 2. Table S7a reports the mathematics coefficient for each science confidence outcome under the four nested specifications summarized in Table 3 of the manuscript, separately by education system. Table S7b reports the proportion of mathematics achievement variance surviving the conditioning, which Reviewer 3 (major comment 2) requested.

Table S7a

Mathematics Coefficient Under Four Nested Conditioning Sets, by System

System	Confidence	M0 zero-order	M1 + own subject	M2 + overall science	M3 + four domains
Austria	Biology	+0.144	−0.185	−0.247	−0.146
Austria	Earth science	+0.177	+0.011	+0.033	+0.082
Austria	Chemistry	+0.198	−0.029	−0.026	−0.013
Austria	Physics	+0.178	+0.018	−0.089	−0.070
Azerbaijan	Biology	+0.112	−0.090	−0.110	−0.095
Azerbaijan	Earth science	+0.148	+0.032	−0.015	+0.010
Azerbaijan	Chemistry	+0.074	+0.026	+0.096	+0.077
Azerbaijan	Physics	+0.186	+0.068	+0.073	+0.071
Cyprus	Biology	+0.230	−0.101	−0.151	−0.082
Cyprus	Earth science	+0.239	−0.024	−0.057	−0.009
Cyprus	Chemistry	+0.277	+0.042	+0.009	+0.032
Cyprus	Physics	+0.281	+0.122	+0.135	+0.144
Czechia	Biology	+0.053	−0.288	−0.392	−0.288
Czechia	Earth science	+0.105	−0.103	−0.142	−0.103
Czechia	Chemistry	+0.183	−0.028	−0.033	−0.045
Czechia	Physics	+0.226	+0.044	−0.009	−0.014
Finland	Biology	+0.180	−0.186	−0.250	−0.142
Finland	Earth science	+0.262	−0.002	−0.062	−0.014
Finland	Chemistry	+0.355	+0.170	+0.212	+0.184
Finland	Physics	+0.328	+0.232	+0.234	+0.193
Georgia	Biology	+0.142	−0.104	−0.182	−0.125
Georgia	Earth science	+0.155	+0.013	−0.077	−0.072
Georgia	Chemistry	+0.241	+0.114	+0.068	+0.058

Georgia	Physics	+0.253	+0.145	+0.135	+0.133
Hungary	Biology	+0.057	−0.359	−0.454	−0.334
Hungary	Earth science	+0.170	−0.112	−0.158	−0.101
Hungary	Chemistry	+0.127	−0.090	−0.114	−0.109
Hungary	Physics	+0.204	+0.063	+0.036	+0.025
Kazakhstan	Biology	+0.107	−0.157	−0.201	−0.142
Kazakhstan	Earth science	+0.176	−0.005	−0.068	−0.033
Kazakhstan	Chemistry	+0.220	+0.176	+0.204	+0.177
Kazakhstan	Physics	+0.216	+0.162	+0.181	+0.174
Lithuania	Biology	+0.084	−0.273	−0.379	−0.285
Lithuania	Earth science	+0.168	−0.093	−0.108	−0.083
Lithuania	Chemistry	+0.278	+0.192	+0.169	+0.163
Lithuania	Physics	+0.225	+0.116	+0.142	+0.134
Morocco	Biology	+0.107	−0.045	−0.115	−0.070
Morocco	Earth science	+0.098	+0.000	−0.115	−0.054
Morocco	Chemistry	+0.242	+0.137	+0.074	+0.092
Morocco	Physics	+0.240	+0.125	+0.082	+0.104
Uzbekistan	Biology	+0.132	−0.049	−0.088	−0.046
Uzbekistan	Earth science	+0.143	+0.092	+0.020	+0.029
Uzbekistan	Chemistry	+0.148	+0.068	+0.081	+0.080
Uzbekistan	Physics	+0.199	+0.117	+0.113	+0.109

Note. M0 is the weighted bivariate association without covariates. M1–M3 include student sex and home educational resources. M3 is the specification used throughout the manuscript. The sign reversal for biology between M0 and M1–M3 occurs in every system.

Table S7b

Variance of Mathematics Achievement Surviving the Conditioning

System	R^2 (mathematics on four science domains)	Residual variance share	Max VIF	Condition number
Austria	0.643	0.357	7.96	6.67
Azerbaijan	0.542	0.458	5.57	5.36
Cyprus	0.616	0.384	7.63	6.46
Czechia	0.666	0.334	6.99	6.17
Finland	0.658	0.342	7.84	6.60
Georgia	0.555	0.445	5.41	5.29
Hungary	0.701	0.299	8.34	6.96
Kazakhstan	0.537	0.463	6.95	6.03
Lithuania	0.704	0.296	9.34	7.26
Morocco	0.478	0.522	4.22	4.51
Uzbekistan	0.502	0.498	5.06	5.17

Note. Computed on the common sample, averaged over the five plausible values, weighted by TOTWGT. The residual share is the proportion of mathematics achievement variance on which the mathematics coefficient is identified.

Section S8. Re-estimation Including France and Portugal

Added in revision in response to Reviewer 2 and Reviewer 3 (major comment 10). All models were re-estimated with France and Portugal retained, giving k = 13 systems.

Table S8

Mathematics-Anchored Associations With France and Portugal Retained (k = 13)

Confidence	β	SE	p	95% CI	I^2	France	Portugal
Biology	−0.163	0.025	0.000	[−0.218, −0.109]	85.1	−0.202	−0.186
Earth science	−0.054	0.022	0.028	[−0.102, −0.007]	72.6	−0.202	−0.186
Chemistry	+0.074	0.023	0.008	[+0.024, +0.125]	72.9	+0.115	+0.128
Physics	+0.098	0.020	0.000	[+0.054, +0.141]	72.5	+0.114	+0.128

Note. Compare the 11-system estimates in Table 4 of the manuscript: biology −.158, earth science −.033, chemistry .066, physics .093. Every conclusion is unchanged and three of the four estimates strengthen. France and Portugal return identical biology and earth science coefficients, which follows directly from the duplication of their paired response blocks and is further evidence of it.

Section S9. Mathematics Content-Domain Anchors by Education System

Added in revision in response to Reviewer 3 (major comment 3). Each TIMSS mathematics content domain was substituted for the overall mathematics score, conditional on the same four science content domains and covariates.

Table S9

Associations Under Alternative Mathematics Anchors, by System

System	Mathematics anchor	Biology	Earth science	Chemistry	Physics
Austria	Overall	−0.146	+0.082	−0.013	−0.070
Austria	Number	−0.141	+0.100	−0.031	−0.051
Austria	Algebra	−0.104	+0.061	+0.037	−0.070
Austria	Geometry and measurement	−0.124	+0.044	−0.053	−0.098
Austria	Data and probability	−0.116	+0.034	−0.024	−0.051
Azerbaijan	Overall	−0.095	+0.010	+0.077	+0.071
Azerbaijan	Number	−0.112	−0.008	+0.082	+0.068
Azerbaijan	Algebra	−0.072	−0.007	+0.103	+0.082
Azerbaijan	Geometry and measurement	−0.079	−0.005	+0.047	+0.072
Azerbaijan	Data and probability	−0.052	+0.043	+0.041	+0.072
Cyprus	Overall	−0.082	−0.009	+0.032	+0.144
Cyprus	Number	−0.053	+0.019	+0.002	+0.177
Cyprus	Algebra	−0.095	−0.045	+0.073	+0.101
Cyprus	Geometry and measurement	−0.088	−0.002	+0.035	+0.106
Cyprus	Data and probability	−0.121	+0.010	−0.035	+0.112
Czechia	Overall	−0.288	−0.103	−0.045	−0.014
Czechia	Number	−0.250	−0.058	−0.009	+0.003
Czechia	Algebra	−0.199	−0.086	−0.012	+0.011
Czechia	Geometry and measurement	−0.226	−0.129	−0.009	+0.035
Czechia	Data and probability	−0.277	−0.081	−0.112	−0.115
Finland	Overall	−0.142	−0.014	+0.184	+0.193
Finland	Number	−0.121	−0.026	+0.174	+0.159

Finland	Algebra	−0.144	−0.008	+0.203	+0.208
Finland	Geometry and measurement	−0.097	−0.026	+0.136	+0.166
Finland	Data and probability	−0.146	−0.023	+0.038	+0.079
Georgia	Overall	−0.125	−0.072	+0.058	+0.133
Georgia	Number	−0.152	−0.058	+0.051	+0.122
Georgia	Algebra	−0.088	−0.051	+0.081	+0.115
Georgia	Geometry and measurement	−0.038	−0.046	+0.049	+0.140
Georgia	Data and probability	−0.073	−0.061	+0.022	+0.066
Hungary	Overall	−0.334	−0.101	−0.109	+0.025
Hungary	Number	−0.288	−0.124	−0.076	+0.019
Hungary	Algebra	−0.322	−0.088	−0.094	+0.045
Hungary	Geometry and measurement	−0.304	−0.101	−0.101	+0.102
Hungary	Data and probability	−0.265	−0.052	−0.105	−0.014
Kazakhstan	Overall	−0.142	−0.033	+0.177	+0.174
Kazakhstan	Number	−0.102	−0.020	+0.170	+0.152
Kazakhstan	Algebra	−0.129	−0.033	+0.157	+0.173
Kazakhstan	Geometry and measurement	−0.106	−0.036	+0.152	+0.142
Kazakhstan	Data and probability	−0.093	−0.000	+0.127	+0.159
Lithuania	Overall	−0.285	−0.083	+0.163	+0.134
Lithuania	Number	−0.253	−0.083	+0.142	+0.127
Lithuania	Algebra	−0.248	−0.011	+0.179	+0.080
Lithuania	Geometry and measurement	−0.276	−0.133	+0.114	+0.138
Lithuania	Data and probability	−0.245	−0.024	+0.076	+0.038
Morocco	Overall	−0.070	−0.054	+0.092	+0.104
Morocco	Number	−0.082	−0.042	+0.081	+0.105
Morocco	Algebra	−0.068	−0.065	+0.080	+0.086
Morocco	Geometry and measurement	−0.051	−0.047	+0.063	+0.090
Morocco	Data and probability	−0.018	−0.022	+0.082	+0.067
Uzbekistan	Overall	−0.046	+0.029	+0.080	+0.109
Uzbekistan	Number	+0.005	+0.072	+0.126	+0.143
Uzbekistan	Algebra	−0.056	+0.020	+0.064	+0.110
Uzbekistan	Geometry and measurement	−0.057	−0.009	+0.028	+0.064
Uzbekistan	Data and probability	−0.094	−0.042	−0.036	+0.050

Note. The biology association is negative in 54 of the 55 system-by-anchor cells, the single exception being the Number anchor in Uzbekistan (+.005). The chemistry association changes sign across anchors in several systems, consistent with its instability across measurement specifications.

Section S10. Anchored Minus Unanchored Differences by Education System

Added in revision in response to Reviewer 1 (comment 2). The difference between the anchored and unanchored cross-path coefficients was formed inside every jackknife replicate and every plausible value before its variance was computed. Pooled values appear in Table 6 of the manuscript.

Table S10

Anchored Minus Unanchored Cross-Path Differences by System

System	Confidence	Achievement	Unanchored	Anchored	Δ	SE
Austria	Biology	Earth science	+0.065	+0.086	+0.020	0.010
Austria	Biology	Chemistry	+0.007	+0.047	+0.040	0.018
Austria	Biology	Physics	−0.301	−0.263	+0.038	0.014
Austria	Earth science	Biology	+0.258	+0.253	−0.006	0.007
Austria	Earth science	Chemistry	−0.021	−0.044	−0.023	0.013
Austria	Earth science	Physics	−0.373	−0.395	−0.023	0.015
Austria	Chemistry	Biology	+0.052	+0.053	+0.001	0.005
Austria	Chemistry	Earth science	−0.021	−0.017	+0.004	0.009
Austria	Chemistry	Physics	−0.100	−0.094	+0.006	0.018
Austria	Physics	Biology	+0.085	+0.090	+0.005	0.005
Austria	Physics	Earth science	−0.073	−0.060	+0.013	0.011
Austria	Physics	Chemistry	+0.312	+0.337	+0.025	0.017
Azerbaijan	Biology	Earth science	+0.007	+0.022	+0.015	0.005
Azerbaijan	Biology	Chemistry	+0.075	+0.097	+0.022	0.008
Azerbaijan	Biology	Physics	−0.102	−0.081	+0.021	0.008
Azerbaijan	Earth science	Biology	+0.076	+0.077	+0.000	0.007
Azerbaijan	Earth science	Chemistry	−0.038	−0.038	+0.000	0.008
Azerbaijan	Earth science	Physics	+0.078	+0.078	+0.000	0.007
Azerbaijan	Chemistry	Biology	−0.038	−0.053	−0.015	0.008
Azerbaijan	Chemistry	Earth science	−0.119	−0.129	−0.011	0.006
Azerbaijan	Chemistry	Physics	−0.011	−0.027	−0.015	0.007
Azerbaijan	Physics	Biology	−0.003	−0.021	−0.017	0.011
Azerbaijan	Physics	Earth science	−0.088	−0.100	−0.012	0.006
Azerbaijan	Physics	Chemistry	+0.119	+0.101	−0.017	0.007
Cyprus	Biology	Earth science	+0.079	+0.084	+0.006	0.007
Cyprus	Biology	Chemistry	−0.093	−0.075	+0.017	0.014
Cyprus	Biology	Physics	−0.070	−0.051	+0.018	0.014
Cyprus	Earth science	Biology	+0.104	+0.104	−0.000	0.005
Cyprus	Earth science	Chemistry	−0.025	−0.025	+0.000	0.013
Cyprus	Earth science	Physics	−0.128	−0.126	+0.001	0.014
Cyprus	Chemistry	Biology	+0.136	+0.132	−0.004	0.005
Cyprus	Chemistry	Earth science	+0.042	+0.038	−0.004	0.004
Cyprus	Chemistry	Physics	−0.041	−0.053	−0.011	0.010
Cyprus	Physics	Biology	−0.021	−0.036	−0.015	0.009
Cyprus	Physics	Earth science	−0.004	−0.016	−0.012	0.007
Cyprus	Physics	Chemistry	−0.019	−0.058	−0.040	0.011
Czechia	Biology	Earth science	+0.025	+0.072	+0.047	0.011
Czechia	Biology	Chemistry	−0.133	−0.013	+0.121	0.015
Czechia	Biology	Physics	−0.109	−0.051	+0.058	0.010
Czechia	Earth science	Biology	+0.081	+0.085	+0.004	0.003
Czechia	Earth science	Chemistry	+0.001	+0.042	+0.041	0.019
Czechia	Earth science	Physics	−0.151	−0.132	+0.020	0.008
Czechia	Chemistry	Biology	−0.114	−0.112	+0.003	0.003
Czechia	Chemistry	Earth science	+0.107	+0.115	+0.008	0.008
Czechia	Chemistry	Physics	+0.049	+0.059	+0.010	0.009
Czechia	Physics	Biology	−0.156	−0.156	+0.000	0.001
Czechia	Physics	Earth science	+0.126	+0.128	+0.002	0.005
Czechia	Physics	Chemistry	+0.142	+0.147	+0.006	0.014
Finland	Biology	Earth science	+0.137	+0.148	+0.011	0.008
Finland	Biology	Chemistry	−0.103	−0.053	+0.050	0.014
Finland	Biology	Physics	−0.233	−0.182	+0.051	0.014
Finland	Earth science	Biology	+0.197	+0.196	−0.000	0.002
Finland	Earth science	Chemistry	−0.061	−0.057	+0.005	0.013
Finland	Earth science	Physics	−0.038	−0.033	+0.005	0.013
Finland	Chemistry	Biology	−0.080	−0.081	−0.001	0.010
Finland	Chemistry	Earth science	+0.145	+0.131	−0.014	0.009
Finland	Chemistry	Physics	+0.005	−0.060	−0.064	0.023
Finland	Physics	Biology	−0.165	−0.166	−0.001	0.010
Finland	Physics	Earth science	+0.124	+0.109	−0.015	0.009
Finland	Physics	Chemistry	+0.287	+0.221	−0.066	0.017
Georgia	Biology	Earth science	+0.098	+0.115	+0.017	0.010

Georgia	Biology	Chemistry	+0.088	+0.110	+0.023	0.008
Georgia	Biology	Physics	−0.101	−0.066	+0.036	0.013
Georgia	Earth science	Biology	+0.090	+0.104	+0.014	0.011
Georgia	Earth science	Chemistry	+0.103	+0.118	+0.015	0.008
Georgia	Earth science	Physics	+0.041	+0.065	+0.024	0.013
Georgia	Chemistry	Biology	+0.001	−0.006	−0.006	0.006
Georgia	Chemistry	Earth science	−0.024	−0.031	−0.007	0.007
Georgia	Chemistry	Physics	+0.221	+0.206	−0.015	0.013
Georgia	Physics	Biology	+0.118	+0.097	−0.020	0.010
Georgia	Physics	Earth science	−0.031	−0.050	−0.019	0.011
Georgia	Physics	Chemistry	+0.033	+0.007	−0.026	0.011
Hungary	Biology	Earth science	−0.134	−0.059	+0.075	0.017
Hungary	Biology	Chemistry	−0.069	+0.035	+0.105	0.022
Hungary	Biology	Physics	−0.149	−0.111	+0.038	0.015
Hungary	Earth science	Biology	+0.071	+0.086	+0.014	0.005
Hungary	Earth science	Chemistry	−0.098	−0.066	+0.032	0.011
Hungary	Earth science	Physics	−0.039	−0.027	+0.012	0.007
Hungary	Chemistry	Biology	−0.020	−0.005	+0.015	0.008
Hungary	Chemistry	Earth science	+0.128	+0.153	+0.024	0.014
Hungary	Chemistry	Physics	−0.122	−0.110	+0.013	0.010
Hungary	Physics	Biology	−0.274	−0.278	−0.004	0.007
Hungary	Physics	Earth science	+0.022	+0.016	−0.006	0.010
Hungary	Physics	Chemistry	+0.341	+0.333	−0.009	0.015
Kazakhstan	Biology	Earth science	+0.125	+0.141	+0.017	0.007
Kazakhstan	Biology	Chemistry	−0.069	−0.003	+0.066	0.016
Kazakhstan	Biology	Physics	−0.234	−0.214	+0.020	0.007
Kazakhstan	Earth science	Biology	+0.120	+0.123	+0.003	0.004
Kazakhstan	Earth science	Chemistry	+0.021	+0.037	+0.017	0.017
Kazakhstan	Earth science	Physics	−0.041	−0.036	+0.005	0.005
Kazakhstan	Chemistry	Biology	+0.019	+0.004	−0.015	0.009
Kazakhstan	Chemistry	Earth science	+0.032	+0.011	−0.021	0.010
Kazakhstan	Chemistry	Physics	−0.008	−0.034	−0.026	0.009
Kazakhstan	Physics	Biology	−0.035	−0.050	−0.014	0.008
Kazakhstan	Physics	Earth science	+0.032	+0.012	−0.020	0.009
Kazakhstan	Physics	Chemistry	+0.041	−0.038	−0.080	0.018
Lithuania	Biology	Earth science	+0.003	+0.066	+0.063	0.015
Lithuania	Biology	Chemistry	−0.074	+0.034	+0.108	0.016
Lithuania	Biology	Physics	−0.104	−0.041	+0.063	0.015
Lithuania	Earth science	Biology	+0.043	+0.047	+0.004	0.004
Lithuania	Earth science	Chemistry	+0.029	+0.060	+0.031	0.018
Lithuania	Earth science	Physics	−0.179	−0.161	+0.018	0.010
Lithuania	Chemistry	Biology	+0.151	+0.140	−0.010	0.008
Lithuania	Chemistry	Earth science	+0.098	+0.062	−0.036	0.008
Lithuania	Chemistry	Physics	−0.032	−0.069	−0.037	0.016
Lithuania	Physics	Biology	−0.053	−0.061	−0.008	0.006
Lithuania	Physics	Earth science	+0.055	+0.027	−0.028	0.009
Lithuania	Physics	Chemistry	+0.009	−0.040	−0.049	0.015
Morocco	Biology	Earth science	+0.049	+0.063	+0.014	0.006
Morocco	Biology	Chemistry	−0.051	−0.040	+0.010	0.005
Morocco	Biology	Physics	+0.064	+0.084	+0.020	0.009
Morocco	Earth science	Biology	+0.275	+0.280	+0.004	0.003
Morocco	Earth science	Chemistry	−0.071	−0.062	+0.009	0.005
Morocco	Earth science	Physics	+0.024	+0.042	+0.017	0.008
Morocco	Chemistry	Biology	+0.093	+0.085	−0.008	0.004
Morocco	Chemistry	Earth science	+0.020	−0.001	−0.021	0.007
Morocco	Chemistry	Physics	+0.118	+0.088	−0.030	0.014
Morocco	Physics	Biology	+0.112	+0.104	−0.008	0.004
Morocco	Physics	Earth science	−0.009	−0.032	−0.023	0.007
Morocco	Physics	Chemistry	+0.098	+0.082	−0.016	0.006
Uzbekistan	Biology	Earth science	−0.092	−0.085	+0.007	0.006
Uzbekistan	Biology	Chemistry	+0.086	+0.103	+0.016	0.013
Uzbekistan	Biology	Physics	−0.109	−0.097	+0.012	0.013
Uzbekistan	Earth science	Biology	+0.232	+0.227	−0.006	0.008
Uzbekistan	Earth science	Chemistry	+0.108	+0.098	−0.011	0.013
Uzbekistan	Earth science	Physics	−0.037	−0.043	−0.006	0.009
Uzbekistan	Chemistry	Biology	+0.281	+0.269	−0.012	0.008
Uzbekistan	Chemistry	Earth science	−0.216	−0.226	−0.010	0.007
Uzbekistan	Chemistry	Physics	−0.061	−0.077	−0.016	0.009
Uzbekistan	Physics	Biology	+0.170	+0.154	−0.016	0.010
Uzbekistan	Physics	Earth science	−0.170	−0.183	−0.013	0.008
Uzbekistan	Physics	Chemistry	+0.104	+0.074	−0.030	0.015

Note. Δ = anchored − unanchored, a difference in signed coefficients. SE is the jackknife repeated replication standard error combined with imputation variance by Rubin's rules.

Section S11. Sampling Covariance of the Mathematics-Anchored Coefficients

Added in revision in response to Reviewer 3 (major comment 1). The ordering probabilities in Table 8 of the manuscript were previously computed by drawing each system's four mathematics-anchored coefficients from independent normal distributions. Because the four coefficients are estimated from the same students in the same multivariate regression, their sampling errors are correlated, and the independence assumption was not tenable. The revised calculation draws from a multivariate normal distribution with the full 4 × 4 sampling covariance matrix.

The covariance was recovered from the replicate-level estimates rather than assumed. Within each plausible value m the jackknife covariance was computed as $C(m) = ½ \Sigma (b_r - b_0)(b_r - b_0)'$ over the replicate estimates b_r, the one-half factor applying because both complementary replicates are formed for every sampling zone. The five matrices were then combined with the between-plausible-value covariance by the matrix form of Rubin's rules, $C = \text{mean}_m C(m) + (1 + 1/M) \text{Cov}_m(b_m)$, with M = 5. Table S11 reports the resulting correlations.

Table S11

Correlations Among the Four Mathematics-Anchored Coefficients Within Each Education System

System	Bio–Earth	Bio–Chem	Bio–Phys	Earth–Chem	Earth–Phys	Chem–Phys
Austria	.48	.15	.29	.50	.61	.81
Azerbaijan	.44	.17	.24	.01	.40	.32
Cyprus	.27	.48	.46	.48	.25	.48
Czechia	−.05	.27	.18	−.41	.50	−.15
Finland	.66	.17	.21	.31	.32	.91
Georgia	.48	.46	.50	.44	.33	.58
Hungary	.41	.52	.22	.47	.18	.15
Kazakhstan	.64	.52	.62	.60	.59	.73
Lithuania	.61	−.01	.08	−.01	.33	.43
Morocco	.76	.47	.44	.49	.41	.83
Uzbekistan	.75	.73	.59	.78	.76	.74

Note. Correlations derived from the combined jackknife and imputation covariance matrix described above. The median absolute correlation is .46, the minimum .01, and the maximum .91. Correlations of this magnitude are large enough that treating the four coefficients as independent materially misstates the ordering probabilities; the consequence is shown in the two probability columns of Table 8 in the manuscript, which report the joint and the independent calculations side by side.
